# CRISPR/dCas12a-mediated activation of *SlPAL2* enhances tomato resistance against bacterial canker disease

**DOI:** 10.1371/journal.pone.0320436

**Published:** 2025-03-26

**Authors:** Diana Marcela Rivera-Toro, Stefan de Folter, Raúl Alvarez-Venegas

**Affiliations:** 1 Center for Research and Advanced Studies of the National Polytechnic Institute (Cinvestav), Unidad Irapuato, Irapuato, Guanajuato, México,; 2 Center for Research and Advanced Studies of the National Polytechnic Institute (Cinvestav), Advanced Genomics Unit, Irapuato, Guanajuato, México; Shiraz University, IRAN, ISLAMIC REPUBLIC OF

## Abstract

Crop protection is essential for maintaining and improving agricultural productivity. While pesticides are commonly used to control pests, they pose several challenges, including environmental harm and health risks. Alternative strategies to pesticides include breeding resistant crop varieties, biological control, and utilizing genome-editing tools like CRISPR/Cas. However, the application of epigenome editing, particularly CRISPR activation (CRISPRa), in plants remains underexplored. Phenylalanine ammonia-lyase (PAL), a key enzyme in the phenylpropanoid pathway, plays a pivotal role in plant defense by producing lignin and other secondary metabolites essential for pathogen resistance. In this study, we engineered tomato plants by fusing the SET-domain of the *SlATX1* coding gene, a histone H3 lysine 4 tri-methyltransferase, to dCas12a, targeting the *SlPAL2* promoter with the aim to increase *PAL2* gene expression. CRISPRa-edited plants demonstrated increased deposition of the H3K4me3 epigenetic mark and significantly upregulated *SlPAL2* expression. This enhanced lignin accumulation and conferred increased resistance to *Clavibacter michiganensis* subsp. *michiganensis* (Cmm) without significant reduction in plant height or fruit yield. Disease resistance was also associated with reduced pathogen load and lesion size, and higher lignin levels persisted even after *SlPAL2* expression declined post-infection. These findings highlight the potential of CRISPRa for reprogramming plant defense responses through targeted histone modifications, offering a sustainable approach for crop improvement. Furthermore, CRISPRa could also be applied to enhance crop resilience in other contexts, such as addressing food security challenges by enhancing productivity.

## Introduction

Crop protection plays a crucial role in preserving and increasing crop productivity. While pesticides are effective for controlling pests, they come with several significant drawbacks, such as adverse effects on human safety, environmental damage, and negative impacts on beneficial organisms [1,2]. To support the growing human population, it is imperative to utilize feasible new strategies for crop production and improved integrated pest management systems. Alternative approaches to the utilization of pesticides comprise the development of new resistant crop cultivars, the employment of biological control agents, the use of elicitors to enhance natural plant defenses [3], and the application of genome engineering tools such as the CRISPR/Cas system. The potential of RNA-programmed Cas proteins for gene-targeting and genome-editing was first published in 2012 [4], and its application in plants was reported in 2013 [5–7]. Since then, numerous studies have showcased the applications of the CRISPR/Cas system in plants, demonstrating its ability to create stable and heritable modifications, such as in tomato plants [8,9], or to engineer quantitative trait variation for crop improvement. Genome editing offers an alternative to conventional plant breeding practices by facilitating the molecular breeding of crops with specific desirable traits. However, studies on plant epigenome editing for gene activation (“CRISPR activation”) are still insufficient. Specifically, there has been little exploration of using engineered Cas12a (defective Cas12a) for the activation of plant defense genes through epigenome editing.

In higher plants, the enzyme phenylalanine ammonia-lyase (PAL) catalyzes the deamination of L-phenylalanine to produce trans-cinnamate, the first step in the multi-branched phenylpropanoid pathway. This pathway is crucial not only for the biosynthesis of phenolic compounds, including flavonoids and lignin, but also for the synthesis of defense-related molecules such as salicylic acid [10,11]. The phenylpropanoid pathway is activated in response to various biotic and abiotic stresses such as pathogen infection [12], mechanical wounding [13], or exposure to elicitors [14]. An important function of this pathway is the production of hydroxycinnamyl alcohols, or monolignols, which are essential precursors for lignin biosynthesis. Lignin is a major structural polymer in plant cell walls that provides structural support, contributes to vascular integrity, and enhances resistance against pathogens [15,16].

In the model plant *Arabidopsis thaliana*, the *PAL* gene family consists of four members (*AtPAL1* to *AtPAL4*) [17], and these genes play key roles in plant defense responses [18]. For instance, infection of Arabidopsis plants with *Pseudomonas syringae* pv. *tomato* (JL1065) has been shown to induce *PAL2* promoter activity in the roots, suggesting that PAL2 contributes to an induced systemic defense response [19]. Studies in tobacco plants (*Nicotiana tabacum L.*) provide further insights into the role of PAL in plant immunity. Tobacco plants overexpressing the bean *PAL2* gene demonstrated increased levels of hydroxycinnamic acid derivatives, including chlorogenic acid, which is associated with enhanced resistance to fungal pathogens like *Cercospora nicotianae* [20]. These transgenic plants also showed increased systemic resistance to tobacco mosaic virus (TMV) infection, further highlighting the essential role of PAL in both local and systemic plant defense responses [20,21].

Tomato phenylalanine ammonia-lyase is encoded by a large multigene family comprising over 20 members in the diploid genome, grouped into at least five different classes (*SlPAL1* to *SlPAL5*; [22]). However, only a subset of the *PAL* genes may be functionally important under normal growth conditions [23–25]. Nevertheless, cultured tomato (*Solanum lycopersicum*) cells exposed to fungal elicitors have shown increased de novo synthesis of enzymes in the prechorismate pathway, as well as elevated levels of *PAL* transcripts [26]. This highlights the importance of the phenylpropanoid pathway and its associated compounds in plant disease resistance. Thus, the phenylpropanoid pathway plays a central role in the plant’s defense response, particularly through the production of lignin and phenolic compounds, which contribute to structural fortifications and antimicrobial activities.

Recently, Tsitsekian et al. [27] investigated the defense responses of tomato plants infected by *Clavibacter michiganensis* subsp. *michiganensis* (Cmm), the causative agent of bacterial canker disease. They identified two distinct phases of defense activation during the infection, occurring at 6 and 12 days post-infection (dpi). The first, or primary defense response, involves the activation of the phenylpropanoid pathway, particularly the PAL enzyme, which is redirected towards the synthesis of coniferyl monolignols. This leads to the accumulation of guaiacyl (G-type) lignin at 6 dpi, which plays an important role in the primary defense response, as defense-induced lignification (lignin deposition in the plant cell walls) is a basal defense mechanism that strengthens cell walls and restricts pathogen spread. In addition to structural reinforcement, lignin also plays a chemical role by being toxic to some pathogens and limiting their enzymatic degradation of plant tissues [28]. The second phase, occurring at 12 dpi, represents a secondary defense response. This phase is marked by downregulation of genes modulating the lignin-mediated defense mechanisms, with a concurrent upregulation of secondary defense-related genes. These genes are involved in more specialized defense pathways, such as those leading to the production of antimicrobial peptides, or other defense-related secondary metabolites [27]. Consequently, the shift from lignification in the early stages of infection to the activation of secondary defense responses at later stages suggests a sophisticated and dynamic defense strategy in plants. Early lignification limits pathogen entrance, while later responses might be adapted to more specific pathogen challenges.

We hypothesized that enhanced expression of the *SlPAL2* gene in tomato (*Solanum lycopersicum*) plants would confer resistance to the highly pathogenic bacterium *Clavibacter michiganensis* subsp. *michiganensis* (Cmm), the causative agent of bacterial canker disease. To test this, we aimed to engineer targeted modifications in the phenylpropanoid metabolic pathway. By activating an early pathway gene using CRISPR activation (CRISPRa), we sought to gain a better understanding of the molecular mechanisms underlying the tomato plant’s response to Cmm. Accordingly, our strategy involved epigenetically editing of the *SlPAL2* gene to induce its expression. Specifically, we fused the catalytic SET-domain of the tomato *SlATX1* coding gene, which possesses histone H3 lysine 4 tri-methyltransferase activity, to the carboxy-terminus of the deactivated CRISPR-associated protein, dCas12a (LbCpf1). This fusion was then directed to the promoter region of *SlPAL2*, facilitating targeted histone modifications that would enhance gene expression. Tomato explants were transformed via biolistics, and the plants were regenerated through somatic embryogenesis. The epigenetically edited plants were subsequently analyzed following Cmm infection to assess their defense responses.

Our results demonstrated that the edited plants exhibited enhanced deposition of the H3K4me3 mark, an epigenetic marker associated with active transcription, at the *SlPAL2* first exonic region. This was accompanied by a significant upregulation of *SlPAL2* transcription. The edited plants also displayed increased tolerance to biotic stress when exposed to Cmm bacterial inoculum, and significantly, showed elevated lignin accumulation. Interestingly, our analysis revealed that the enhanced resistance to Cmm did not come at the expense of agronomic traits. The epigenetically modified plants maintained normal growth and fruit development, suggesting that the modifications did not result in any fitness costs—a crucial consideration for practical agricultural applications. This work underscores the potential of epigenetic editing as a distinctive approach for enhancing plant defense mechanisms. By reprogramming gene expression through histone modifications, we can create crops that are more resilient to pathogens without negatively impacting yield or quality. The use of CRISPRa for targeted manipulation of the phenylpropanoid pathway could be a powerful tool in crop improvement programs. Future applications of these techniques have the potential to mitigate significant economic losses caused by plant diseases and contribute to more sustainable agricultural practices by improving crop resilience and sustainability.

## Materials and methods

### CRISPRa vectors

For vector construction we have implemented the procedures and vectors described by [29]. Briefly, a 1000 bp region upstream of the coding sequence of the *SlPAL2* gene in *Solanum lycopersicum* (Gene ID: 101249824, GenBank accession: NM_001320601, Solgenomics: Solyc05g056170.2) was selected for CRISPR RNA (crRNA) design. Candidate crRNAs for CRISPR/dCas12a (LbCpf1, PAM: 5’-TTTV-3’) binding were identified using online tools such as CRISPR-P 2.0 [30] and CHOPCHOP [31]. No potential off-targets were predicted (see S1 Table for a detailed analysis). Three crRNAs were chosen based on their location within the first 300 bp upstream of the transcription start site (TSS), ensuring that no cis-regulatory elements—such as the TATA and CAAT boxes, or the TSS itself—were disrupted. A tandem array of the three crRNAs (referred to here as crArray) was designed following the method of [32], taking advantage of Cas12’s ability to process pre-crRNAs without the need for RNase III for crRNA release. Each crRNA was separated by direct repeat (DR) sequences specific to LbCpf1 (Cas12a from *Lachnospiraceae* bacterium). This array was synthesized as a double-stranded DNA fragment (Synbio Technologies, USA) and subcloned into the BsaI sites of the p143-L2 vector [29].

The p143-L2 vector, the entry vector containing dCas12a fused to the catalytic SET domain of ATX1 (p233-SETX), and the destination vector for recombination (CT2H or p203-GFP-Hyg) were generated as previously described [29]. For recombination, each vector was linearized as follows: 100 ng of the p143-L2-crRNA array vector were digested with EcoRV, 100 ng of the p233-SETX vector were digested with HpaI, and 200 ng of the p203-GFP-Hyg destination vector were digested with BglII. The Gateway LR Clonase II Enzyme Mix kit (Invitrogen, USA) was used for the recombination reaction following the manufacturer’s instructions to obtain the FS1H-PAL2 vector (dCas12 + SET+crRNA-PAL2). For the control vector, the above-mentioned methodology was followed, but with the p143-L2 vector lacking crRNA, resulting in the SL0H vector (dCas12 + SET+ΔcrRNA). The empty CT2H (p203-GFP-Hyg) vector was also used as control. For detailed vector descriptions and DNA sequences, see S1 Fig and S2 Table.

### Tomato transformation and plant regeneration

The protocols for cotyledon explant preparation, microprojectile bombardment, and plant regeneration were conducted following the methods outlined by [29]. Briefly, *Micro-Tom* tomato seeds (Moles Seeds, cat. # VTO325) were surface-sterilized, manually scarified, and placed on Murashige and Skoog (MS) basal medium (Sigma-Aldrich, Cat. # M5519), supplemented with activated charcoal (3 g/L) and Gelrite (3 g/L; Sigma-Aldrich, Cat. # G1910). The seeds were incubated in a Percival growth chamber at 22°C under long-day conditions (16 h light/8 h darkness) with an irradiance of 50 μmol m⁻² s⁻¹, provided by fluorescent T8 Phillips P32T8/TL850 lamps. After 8 days, cotyledons were excised from the embryonic axis, and 3-mm-long cotyledon explants were sub-cultured in MS-BK2iP medium containing 5% sucrose and 5 g/L Gelrite (osmotic treatment medium) for 24 hours.

Each experimental unit, or bombarded plate, contained 20 cotyledon explants (3 mm²), with 9 plates used for each CRISPRa construct. Microcarriers were prepared using 100 ng of plasmid DNA precipitated onto gold microprojectiles (1.0 µm diameter), and a Bio-Rad PDS-1000/He particle delivery system was employed to bombard the explants, with the abaxial side facing upwards. Control samples were bombarded with either empty vectors or without DNA. Following bombardment, the explants were maintained for 5 days on MS-BK2iP medium, containing 3% sucrose and 3 g/L Gelrite, without antibiotics. Subsequently, the explants were transferred to MS-BK2iP + Hyg medium, supplemented with 3% sucrose, 3 g/L Gelrite, and 10 mg/L hygromycin (pH 5.8). Explants underwent four incubation cycles, each lasting two weeks, in a growth chamber at 22°C under long-day conditions with an irradiance of 50 μmol m⁻² s⁻¹. Next, embryogenic structures were dissected and individually sub-cultured onto fresh selective G92iP medium to induce pro-embryogenic mass formation. Sub-culturing occurred at three-week intervals, three times. Germinated embryos were then transferred to clear plant tissue culture glass bottles for rooting and elongation, where they were maintained for 60 days under the same long-day light cycle. Finally, fully developed individual plants were transferred to 0.7 L plastic pots containing Sunshine Mix #3 potting mix (Sun Grow Horticulture, USA) and grown in a greenhouse under a 14-hour photoperiod with average temperatures ranging from 18–25°C.

### Plant genotyping and propagation

Confirmation of T-DNA insertion was carried out following the protocol described by García-Murillo et al. [33]. Briefly, genomic DNA was extracted from regenerated plants, and the presence of the transgene was confirmed using PCR with specific primers for the 35S CaMV sequence and dCas12 + SET+crRNA+Attb2 region (35S-F 5’-tccttcgcaagacccttc-3’′, 35S-R: 5’-ccttatctgggaactactcacac-3’; dCas12-F: 5’-ttatataccggcgtggcttac-3’, dCas12-R: 5’- accactttgtacaagaaagctg-3’). Plants testing positive for the transgene were propagated, and seeds were collected from these plants. The T1 seeds were collected and germinated under sterile conditions on MS medium [34], and seedlings were later transferred to 0.7 L plastic pots containing Sunshine Mix #3 substrate (Sun Gro Horticulture, USA; sungro.com/professional-product/sunshine-mix-3/). The plants were grown in a greenhouse in Guanajuato, Mexico (101°09′01″ W, 20°30′09″ N; 1730 masl), under natural daylight conditions (14 hours of light and 10 hours of darkness), with an average temperature range of 20–25°C. Prior to infection assays, the presence of the transgene was reconfirmed by PCR to ensure stable inheritance. Throughout the growing period, plants were watered twice a week and fertilized once a week using a combination of Hydrospeed® CaB Max (N:Ca:B, 15:26:0.2) and Ferviafol® (N:P:K:, 20:30:10) to support healthy growth and development.

### Bacterial strain and growth conditions

*Clavibacter michiganensis* subsp. *michiganensis* (Cmm) strain AcR42, kindly provided by Dr. Ángel Gabriel Alpuche Solís (IPICYT, Mexico), was cultured following the protocol outlined by García-Murillo et al. [33]. In brief, the inoculum suspension was prepared by selecting single colonies from a solid SCM medium [35] and growing the bacteria in 100 mL of liquid 802 medium (polypeptone 10 g/L, yeast extract 2 g/L, MgSO₄·7H₂O 1 g/L, pH 7) at 28°C. After 48 h, the bacterial cells were pelleted by centrifugation (3500 g, 8 min, 4°C, repeated twice using a J2-MC Beckman Coulter centrifuge with a JA-14 rotor). The pellet was washed and resuspended in 10 mL of 10 mM MgCl₂. The bacterial concentration was adjusted to 10⁸ cfu mL ⁻ ¹ by serial dilution to reach an OD_600_ of 0.12, as measured by a spectrophotometer (SV1600 VIS, Azzota Co.). This prepared suspension was used as the inoculum for further experiments.

### Pathogen infection by infiltration

Pathogen infiltration, performed three weeks after germination, followed the method described by García-Murillo et al. [33]. The Cmm bacterial suspension (OD_600_ of 0.12 in 10 mM MgCl₂) was loaded into a syringe without a needle. The syringe plunger was then pressed gently against the abaxial surface of the leaf while applying light counter-pressure to the adaxial side to ensure proper infiltration. One infiltration point per leaf (1 cm² each) was used to expand the infiltration area. Control plants were not infected. Susceptibility to Cmm was assessed by measuring the colony-forming units (CFUs) and determining lesion size (see below).

### f.Plant disease severity assessment

Bacterial growth was assessed following established protocols [33,36]. Briefly, nine days post-infection, a leaf disk (1 cm²) adjacent to the infection sites was excised, washed, and homogenized in 10 mM MgCl₂. The homogenates were plated in a 1:100 dilution, on solid 802 medium and incubated at 28°C for 48–72 hours. Colony-forming units (CFUs) were counted from three plates for each biological replicate, and bacterial growth of Cmm was confirmed by transferring 10% of the colonies to semi-selective SCM medium. Additionally, the percentage of leaf lesion area was calculated on leaves infected with Cmm using the R package “Plant Image Analysis -Pliman-” [37]. By providing a color palette to segment the background, the lesions and the healthy leaf are analyzed. Settings involve establishing a “palette” based on the pictures of the leaves that are assessed. In this procedure, the first segment corresponded to healthy tissue, the second represented the lesions, and the third segment corresponded to the background. For this approach, the function fits a general linear model (binomial model) to the RGB values of the image [37].

### Gene expression analysis

RT-qPCR was conducted following the protocol described by Martínez-Aguilar et al. (2016). The data were analyzed using the relative quantification method, specifically the 2^ − ΔΔCT^ method [38]. Expression levels of the *SlPAL2* gene (PAL2-F 5’-agacgtgactgtgcaactatc-3’, PAL2-R 5’-ctatcagtcccatttctcatccc-3’) were normalized to the endogenous control gene *SlLSM7* (SlLSM7-F 5’-gtggaagacaagtggttggaacac-3’, SlLSM7-R 5’-cgtctggctgaacaaaaggattgg-3’) and compared to the expression levels in control samples. All experiments included three technical replicates for each sample and were performed on three independent biological replicates (three distinct clones per line) to ensure accuracy and reproducibility.

### Chromatin immunoprecipitation

Chromatin isolation and immunoprecipitation (ChIP) were performed following the protocols outlined by Martínez-Aguilar et al. [39] and García-Murillo et al. [33], with minor modifications. Leaf tissue samples (200 mg) were fixed with 1% formaldehyde to cross-link proteins and DNA. Chromatin was then isolated and digested with 1 U of micrococcal nuclease (Thermo Scientific cat. #88216) at 37°C for 25 minutes, generating DNA fragments of approximately 250 bp (S2 Fig). The samples were centrifuged at 13,000 rpm for 10 minutes at 4°C, and the resulting digested chromatin was collected and stored at − 80°C until further use. For each immunoprecipitation, aliquots of the chromatin (diluted 10-fold) were processed using the EZ-Magna ChIP kit (Millipore cat. #17-408) along with Magna ChIP Protein A +  G Magnetic Beads (Millipore cat. #16-663), following the manufacturer’s protocol. Immunoprecipitation was carried out overnight at 4°C using 5 μl of anti-trimethyl-histone H3 lysine 4 (H3K4me3) antibody (Millipore cat. #17-614). The bead-antibody/chromatin complex was separated using a Magna Grip Rack (Millipore cat. #20-400), washed, and the protein-DNA complexes were eluted. Crosslinks were reversed, and the DNA was purified for subsequent analysis. An aliquot of the initial digested chromatin was used as an input control after purification. ChIP experiments were performed independently in duplicate (technical replicates) from two biological replicates. PCR amplification was carried out using the Maxima SYBR Green/ROX qPCR Master Mix (Thermo Fisher Scientific) on a Bio-Rad C1000 Thermal Cycler. Primers were designed to amplify putative nucleosomal regions in the first exon: N + 1-F 5′-gcgttaaggctcaacaacaa-3’, N + 1-R 5’- cacgtctttacctgactgtgc-3’ (primer set amplifies region + 45 to + 235 nt); N + 2-F 5’-agacgtgactgtgcaactatc-3′, N + 2-R 5’-ctatcagtcccatttctcatccc-3′ (primer set amplifies region + 238 to + 330 nt). The ChIP-qPCR data was normalized to the input sample (representing 1% of starting chromatin) using the Percent Input method: % Input =  2^[(Cq(IN) −  Log2(DF)) −  Cq(IP)] *  100, as described by Solomon et al. [40].

### Lignin quantification by thioglycolic acid (TGA) method

Lignin content in tomato plants was measured following the protocols of Wang et al. [41] and Dampanaboina et al. [42], with slight modifications. Systemic leaves (1 g) from infected and non-infected tomato plants were powdered in liquid nitrogen and homogenized in 99.5% ethanol. The homogenate was centrifuged at 12,000 × g for 20 minutes at 4°C, and the resulting pellet was air-dried at room temperature for 24 hours. Next, 20 mg of the dried sample was transferred to a 2 mL centrifuge tube, mixed with 0.1 mL of thioglycolic acid (Sigma-Aldrich, cat #T3758) and 1 mL of 2 M HCl, and incubated at 80°C for 3 hours. After cooling on ice, the sample was centrifuged at 14,000 × g for 10 minutes at 4°C. The pellet was washed with distilled water, centrifuged again, resuspended in 1 mL of 1 M NaOH, and gently mixed at 37°C for 18 hours. Following centrifugation at 14,000 × g for 10 minutes, the supernatant was transferred to a new microtube, mixed with 1 mL of concentrated HCl, and the lignin thioglycolic acid was allowed to precipitate at 4°C for 6 hours. After a final centrifugation, the sediment was dissolved in 1 mL of 1 M NaOH, and the absorbance of the solution was measured at 280 nm against a NaOH blank. The lignin content was expressed on a dry weight basis, and a calibration curve was generated using commercial alkali lignin (Sigma-Aldrich, cat #370959) as a standard.

### Agronomic characteristics

We monitored plant height biweekly, along with fruit number and weight, in both edited and control plants to assess the impact of tomato genome editing on these agronomic traits. These characteristics were evaluated in both infected and non-infected plants for comparison.

### Statistical analysis

The study data were analyzed using two statistical approaches in GraphPad Prism 8.0.1 (https://www.graphpad.com). (a) One-Way ANOVA: this method was applied when the data satisfied the classical assumptions of analysis of variance, such as normality and homogeneity of variances; and (b) Welch’s One-Way ANOVA: for data that violated the assumption of homogeneity of variances, Welch’s ANOVA—a robust variant of ANOVA—was used. This method provides reliable results in the presence of heteroscedasticity (unequal variability among groups), reducing the risk of biased or inaccurate conclusions.

Following both One-Way ANOVA and Welch’s ANOVA, Dunnett’s multiple comparisons test was conducted to compare each experimental group with the control group while maintaining control of type I error. By employing these complementary methods based on the specific characteristics of the data, the analysis ensured robustness and validity, minimizing errors and accurately capturing differences between experimental and control groups.

To analyze plant height measurements collected at multiple time points, a two-way ANOVA was performed, followed by Tukey’s multiple comparisons test to assess differences between groups over time.

## Results

### Generation of edited tomato plants via somatic embryogenesis

**Fig 1:**
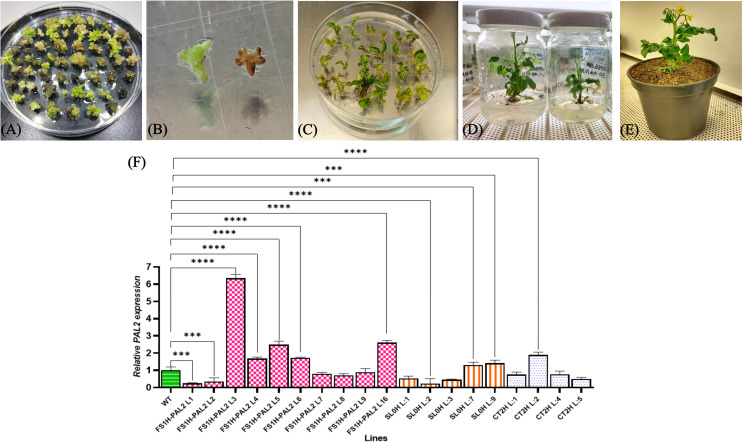
Somatic embryogenesis and plant regeneration process. (A) pro-embryogenic masses transformed with the CRISPR-dCas12a expression vectors, (B) individualization and selection of somatic embryos on hygromycin-containing medium, (C) edited plant regeneration, (D) edited plant elongation and rooting, (E) acclimatization of edited plants, (F) transcript levels of *SlPAL2* in single parental epigenetically edited tomato plants (with three technical replicates of each PCR reaction). Data represents mean ±  SD. Statistical analysis was performed by using Welch’s one-way ANOVA followed by Dunnett’s multiple comparisons test. Statistically significant differences are indicated as follows: **p** <  0.005, * ; **p** <  0.01, **, **p** <  0.001, ***; **p** <  0.0001, ****; the absence of significance bars indicate that no significant (ns) differences were detected.

To generate CRISPRa-edited tomato plants, cotyledon explants from tomato cv. Micro-Tom were cultured for 24 h on MS-BK2iP (high osmotic medium), followed by biolistic transformation with CRISPRa constructs (FS1H-PAL2 or dCas12 + SET + 3crRNA-PAL2; SL0H or dCas12 + SET-ΔcrRNA; and CT2H empty vector or p203-GFP-Hyg). After transformation, the explants were maintained for another 24 hours on the same medium, then transferred to a non-osmotic medium without antibiotics for 5 days. Subsequently, they were moved to a selective medium until pro-embryogenic masses developed (four rounds of selection, each lasting two weeks; Fig 1A). Once individual embryogenic structures were formed, they were dissected and sub-cultured on G92iP medium, incubated at 25°C with a 12/12 h photoperiod and an irradiance of 50 μmol/m − 2 s − 1 (Fig 1B; [29]). After 30-45 days, embryos started to develop roots and shoots (Fig 1C). Plantlets were then sub-cultured for 45 days into clear plant tissue culture glass jars with fresh medium (Fig 1D). Following the protocol described by García-Murillo et al. [33], mature healthy plants were genotyped. As shown in S3 Fig, PCR primers amplified either the CaMV35S promoter fragment (1.26 kb) or the dCas12 + SET+crRNA+Attb2 region (2.5 kb) in FS1H-PAL2 edited plants. SL0H and CT2H control plants (lacking gRNAs or with empty vectors, respectively) only amplified the CaMV35S fragment. Positive T0 edited plants were selected for further experiments. These plants were transferred to soil, acclimatized, and grown to maturity (Fig 1E). To assess the efficiency of the CRISPRa protocol, *SlPAL2* expression levels were measured in confirmed T0 edited clones from the different lines. Fig 1F shows that FS1H clones L3, L5, and L16 exhibited the highest *SlPAL2* expression levels (6.5, 2.5, and 2.6-fold increases, respectively), while the CT2H negative control line, including clones CT2H-L1, CT2H-L4, and CT2H-L5, showed no statistically significant changes in *SlPAL2* expression. Similarly, SL0H clones 1, 2, and 3 exhibited no statistically significant expression changes and were selected for further analysis. Seeds from three representative clones per line were collected for further investigation into targeted epigenetic reprogramming and regulation of the *SlPAL2* gene in response to plant-pathogen interactions.

### Pathogen infection, *PAL2* expression and plant disease assessment

To analyze the CRISPRa-edited tomato plants´ resistance to pathogen infection, two experiments (biological repetitions) were conducted, involving four lines (WT, CT2H, FS1H-PAL2, SL0H) in each trial. Each line had three clones (independent transformation events), except for the WT, which was a single clone, with three plants per clone per time point (S3 Table). The first trial was conducted during the winter period (October to February), while the second trial took place in the spring (April-September). Despite both trials being conducted in the same greenhouse with similar conditions of humidity, luminosity, and temperature, small variations in plant growth and response were observed.

**Fig 2:**
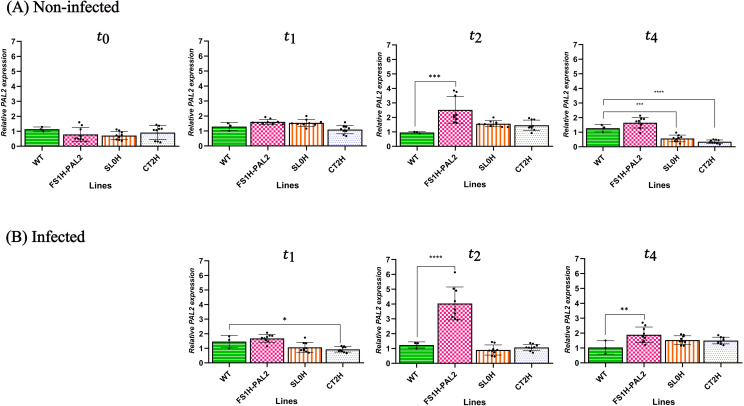
Transcript levels of *SlPAL2.* Transcript levels of *SlPAL2* in edited tomato plants in non-infected plants (A, upper row) and infected plants (B, lower row) at different time points (days post-infection, dpi). Foliage leaves from 3-weeks-old plants were taken at 24 h before Cmm (t_0_), 1 dpi (t_1_), 3 dpi (t_2_), and 60 dpi (t_4_). Data were normalized to the *SlLSM7* reference gene (based on the 2^(-ΔΔCt) method; [38]). Data represent mean ±  SD from three independent clones (n = 3) with three technical replicates per clone (except for the WT line, for which a single representative dataset with three technical replicates is shown). Statistical analysis was performed using one-way ANOVA followed by Dunnett’s multiple comparisons test. Statistically significant differences are indicated as follows: **p** <  0.005, * ; **p** <  0.01, **, **p** <  0.001, ***; **p** <  0.0001, ****; the absence of significance bars indicate that no significant (ns) differences were detected.

To evaluate the susceptibility or enhanced resistance of epigenetically edited tomato plants to *Clavibacter michiganensis* subsp. *michiganensis* (Cmm) infection, samples were collected both before (t_0_ =  24 hours prior to infection) and after infection (t_1_ =  24 hours after infection, t_2_ =  3 days after infection, t_3_ =  9 days after infection, t_4_ =  60 days after infection). The abaxial surface of each leaf was infiltrated with the CmmAcR42 strain at a concentration of 1 x 10⁸ CFU/mL. One infiltration point was used per leaf, and two leaflets per plant were inoculated three weeks post-germination (control plants were not infiltrated). Samples from systemic leaves were collected at 1, 3, and 60 days after infection (t_1_, t_2_, and t_4_); control plant samples were taken at the same time points. *SlPAL2* gene expression was determined using RT-qPCR. As shown in Fig 2, non-inoculated control plants (SL0H, CT2H, WT) exhibited no significant changes in enhanced gene expression at t_0_, t_1_, t_2_, and t_4_. However, in the non-infected edited plants, *SlPAL2* transcript levels were increased at t_2_ and t4 (3.5- and 2.8-fold increases, respectively), suggesting that the edited plants were upregulating endogenous *SlPAL2* expression, although expression declined over time (Fig 2A). After Cmm infection, transcript levels were measured at t_1_, t_2_, and t_4_ (1, 3, and 60 days after infection, respectively). As shown in Fig 2B, edited plants challenged with the pathogen displayed enhanced *SlPAL2* transcript levels 72 hours post-infection, with increases ranging from 3.2- to 5-fold in FS1H-PAL2 plants (normalized with *SlLSM7*), followed by a down-regulation over time (as it happens in the pathosystem *Solanum tuberosum - Rhizoctonia solani* [43]). In contrast, the expression of *SlPAL2* in infected control plants remained largely unchanged across all time points, with no statistically significant differences observed between the lines.

**Fig 3:**
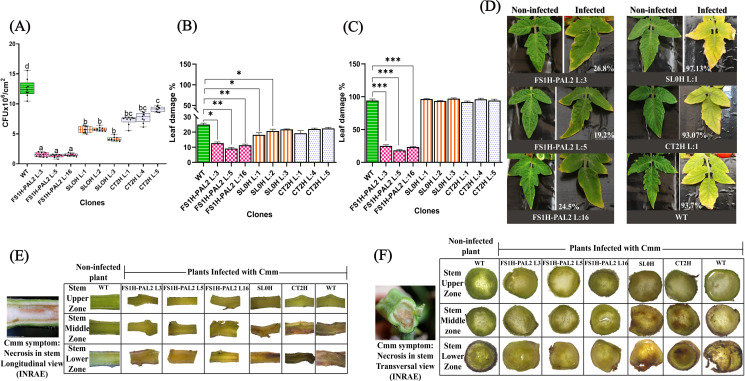
Colony forming units and lesion development in tomato plants after inoculation with CmmAcR42. **(A)** Colony Forming Unit (CFU) count in leaves taken 9 days after Cmm infection. **(B)** Disease severity as percentage of leaf damage in tomato leaves 9 days after infection. **(C)** Disease severity as percentage of leaf damage in tomato leaves 120 days after infection. For (A) a Tukey’s test was performed at α=0.01, with n = 3 biological replicates, each with three technical replicates (data represents mean ±  SEM); for (B) and **(C)**, statistical analysis was performed from three biological replicates (n = 3), using Welch’s one-way ANOVA followed by Dunnett’s multiple comparisons test. Statistically significant differences are indicated as follows: **p** <  0.005, * ; **p** <  0.01, **, **p** <  0.001, ***; **p** <  0.0001, ****; the absence of significance bars indicates that no significant (ns) differences were detected (data represents mean ±  SD). **(D)** Phenotypes of non-infected and infected tomato leaves 120 days after infection. **(E)** Cmm symptomatology in upper, middle and lower stem regions from longitudinal sections from distinct infected tomato plants (reference image taken from INRAE, [44]). **(F)** Cmm symptomatology in upper, middle and lower stem from transversal sections from distinct infected tomato plants (reference image taken from INRAE, [44]).

Next, disease progression was evaluated by quantifying the total number of colony-forming units (CFUs) in leaves of edited plants infected with the pathogen. As shown in Fig 3A, nine days post-infection (t_3_), CFU counts were significantly lower in FS1H-PAL2 edited plants (ranging from 1 to 2 ×  10⁶ CFU per 1 cm^2^ of infected tissue) compared to control plants (e.g., 1.2 ×  10^7^ CFU in WT plants and 5 ×  10^6^ CFU in SL0H plants). This indicates that bacterial populations were substantially reduced in the leaves of edited plants exposed to the pathogen compared to the control plants. Additionally, lesion size (the total chlorotic and necrotic leaf area relative to the total leaf area) was assessed in leaves challenged by the pathogen at 9 and 60 days post-infection using the *pliman* plant image analysis package run in R version 4.4.1 [37]. As seen in Fig 3B, control plants showed signs of chlorosis and necrosis nine days post-infection (around 20% in lesion size), whereas FS1H-PAL2 edited plants remained green and healthy (10% lesion size). Two months post-infection (60 dai; Fig 3C-D), the lesion size in WT, CT2H, and SL0H plants ranged from 93% to 97%, while in FS1H-PAL2 plants, the lesion size was significantly lower, around 23.5%. This indicates that epigenetically edited plants exhibited a significant reduction in disease symptoms and enhanced resistance to Cmm infection compared to non-edited plants. Three months after infection (90 dai), stem canker development was observed in the stem of control plants. Cross-sections (both longitudinal and transverse) revealed yellow to brownish pith in control plants (Fig 3E), with the most severe damage in the lower stem zones. Necrosis and extensive damage to the xylem, sclerenchyma, cortex, and dermal, ground, and vascular tissues were evident (Fig 3F). In contrast, FS1H-PAL2 edited plants displayed few to no symptoms despite harboring variable pathogen populations in the cortex, although the pathogen remained capable of spreading. Non-infected control plants showed no vascular abnormalities. In summary, FS1H-PAL2 epigenetically edited plants showed increased *SlPAL2* transcriptional expression (ranging from 3.2- to 5-fold expression increase), and presented a substantial reduction of disease symptoms in locally infected leaves and enhanced resistance to the disease caused by Cmm (CFUs). Our results are consistent with the hypothesis that directed CRISPRa to the promoter region of the *SlPAL2* gene enhanced its transcriptional activation and protected tomato plants against severe Cmm infection.

**Fig 4:**
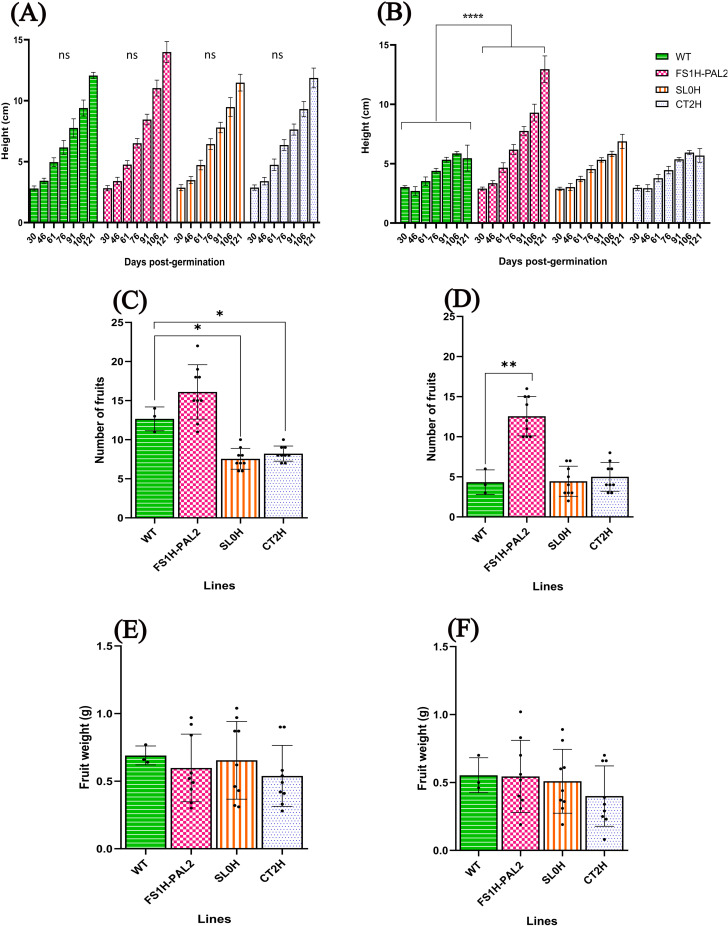
Agronomic characteristics in edited tomato plants. Plant height at different time points (days post-germination): (A) non-infected plants, (B) plants infected with Cmm 10 − 8 CFU mL − 1. Number of fruits: (C) non-infected plants, (D) plants infected with Cmm 10 − 8 CFU mL − 1. Fruit weight: (E) non-infected plants, (F) plants infected with Cmm 10 − 8 CFU mL − 1. For A-B, statistical analysis was performed using two-way ANOVA followed by Tukey’s multiple comparisons test. Statistically significant differences are indicated as follows: **p** <  0.005, * ; **p** <  0.01, **, **p** <  0.001, ***; **p** <  0.0001, ****; data represents mean ±  SD. For C-F, statistical analysis was performed using Welch’s one-way ANOVA followed by Dunnett’s multiple comparisons test, based on six biological replicates (n = 6) (except for the WT line, for which a single representative dataset with three technical replicates is shown). Statistically significant differences are indicated as follows: **p** <  0.005, * ; **p** <  0.01, **, **p** <  0.001, ***; **p** <  0.0001, ****; data represents mean ±  SEM. The absence of significance bars indicates that no significant (ns) differences were detected.

To assess the effect of CRISPRa editing on agronomic traits, we evaluated plant height (measured biweekly), fruit numbers, and fruit weight in both infected and non-infected plants. As shown in Fig 4A, there were no statistically significant differences in plant height among non-infected lines. However, after infection, control plants (SL0H, CT2H, and WT; Fig 4B) showed reduced height, while FS1H-PAL2 plants continued to grow normally. Interestingly, non-infected FS1H-PAL2 plants produced more fruits (16 fruits per plant) compared to SL0H and CT2H control plants (8 fruits per plant; Fig 4C). Although fruit productivity declined across all lines following Cmm infection, all infected control plants still had significantly fewer fruits than FS1H-PAL2 edited plants (Fig 4D). In contrast, there were no statistically significant changes in fruit weight between infected and non-infected plants, nor among the different lines (Fig 4E-F).

**Fig 5:**
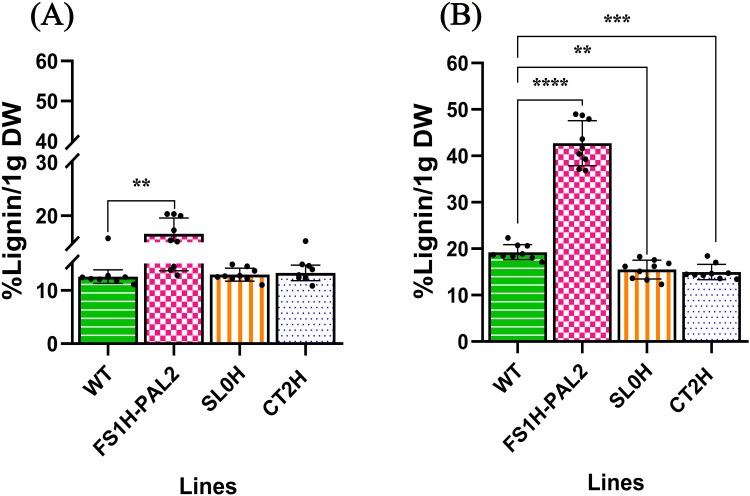
Quantification of lignin contents. Quantification of lignin contents in leaf tissue to assess the effect of *PAL2* gene activation on lignin deposition 120 days post-infection. **(A)** Non-infected plants and (B) plants infected with Cmm 10 − 8 CFU mL − 1. Statistical analysis was performed using Welch’s one-way ANOVA followed by Dunnett’s multiple comparisons test, based on three biological replicates (n = 3) each with three technical replicates. Statistically significant differences are indicated as follows: **p** <  0.005, * ; **p** <  0.01, **, **p** <  0.001, ***; **p** <  0.0001, ****; data represents mean ±  SEM. The absence of significance bars indicates that no significant (ns) differences were detected.

Considering that phenylalanine ammonia-lyase (PAL) is the first committed enzyme in the phenylpropanoid pathway, responsible for the production of lignin, lignans, and flavonoids, and that lignin deposition is a critical defense mechanism in plants against pathogenic attacks [45], we measured lignin content in both non-infected and infected adult tomato plants at 120 days post-infection (Fig 5). Our findings revealed that, in non-infected plants, the highest lignin levels were observed in FS1H-PAL2 edited plants (16.6% lignin content), compared to control plants (SL0H 12.9%, CT2H 13.2%, and WT 12.6%; Fig 5A). Similarly, in infected plants, FS1H-PAL2 edited plants showed the highest lignin content (42.7%). This represents a 2.6-fold increase in lignin accumulation after pathogen infection (from 16.6% to 42.7%). In contrast, lignin content in control plants showed only a modest increase after infection (SL0H 15.5%, CT2H 14.9%, and WT 19.2%; Fig 5B). This suggests that lignin accumulation in edited plants, as a structural component that enhances mechanical support, water transport, and responses to biotic and abiotic stresses [41], plays a vital role in both plant growth (as shown for plant height, Fig 4A) and defense against Cmm, as indicated by *SlPAL2* transcript levels, disease severity, and symptom reduction.

Seeds from all plants were collected and used for a biological repeat to further investigate the targeted epigenetic reprogramming and the regulation of the disease-responsive *PAL2* gene following plant-Cmm interactions.

### Experimental replication, ChIP assay and lignin contents

**Fig 6:**
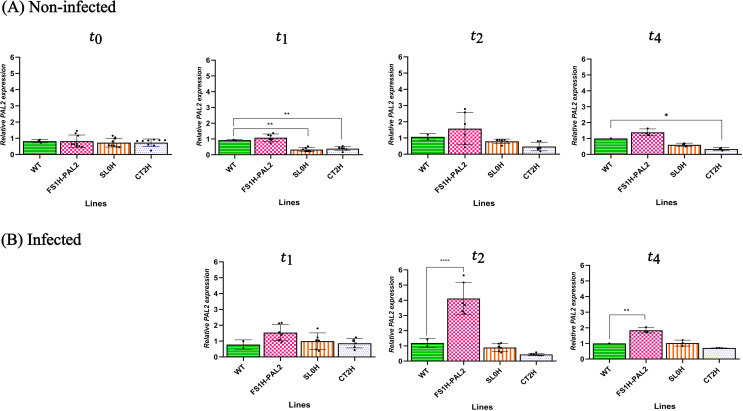
Transcript levels of *SlPAL2.* Transcript levels of *SlPAL2* in edited tomato plants in non-infected plants (A, upper row) and infected plants (B, lower row) at different time points (days post-infection, dpi). Foliage leaves from 3-weeks-old plants were taken at 24 h before Cmm (t_0_), 1 dpi (t_1_), 3 dpi (t_2_), and 120 dpi (t_4_). Data were normalized to the *SlLSM7* reference gene (based on the 2^(-ΔΔCt) method; [38]). Data represent mean ±  SD from three independent clones with three technical replicates per clone (except for the WT line, for which a single representative dataset with three technical replicates is shown). Statistical analysis was performed using one-way ANOVA followed by Dunnett’s multiple comparisons test. Data represents mean ±  SEM. Statistically significant differences are indicated as follows: **p** <  0.005, * ; **p** <  0.01, **, **p** <  0.001, ***; **p** <  0.0001, ****. The absence of significance bars indicates that no significant (ns) differences were detected.

As mentioned above, a second trial was conducted from the April-September term. To further investigate the role of *SlPAL2* activation in response to Cmm infection, seeds were germinated (S3 Table), and plants were genotyped again (S4 Fig). Leaves from 21 days-old plants were infiltrated with the same pathogen concentration as before (1 x 10^8^ CFU/mL). *SlPAL2* expression levels were measured both before and after Cmm infection. As shown in Fig 6A, *SlPAL2* expression in FS1H-PAL2 plants prior to infection was not significantly different than control plants, although transcript levels did show a net increase at t_2_. After infiltration, samples from systemic leaves were collected at 1, 3 and 60 days after inoculation (t_1_, t_2_, t_4_). Samples from healthy non-infected plants were taken at the same times to assess *SlPAL2* expression. As shown in Fig 6B, edited FS1H-PAL2 plants challenged with Cmm exhibited a significant increase in *SlPAL2* transcript levels at t_2_ (72 hours post-infection), with a 3.2- to 5.5-fold increase, followed by a reduction over time (2-fold increase at t_4_) (Fig 6B). In contrast, *SlPAL2* expression in infected control plants remained largely unchanged across all time points, with no statistically significant differences observed between the lines (Fig 6A).

**Fig 7:**
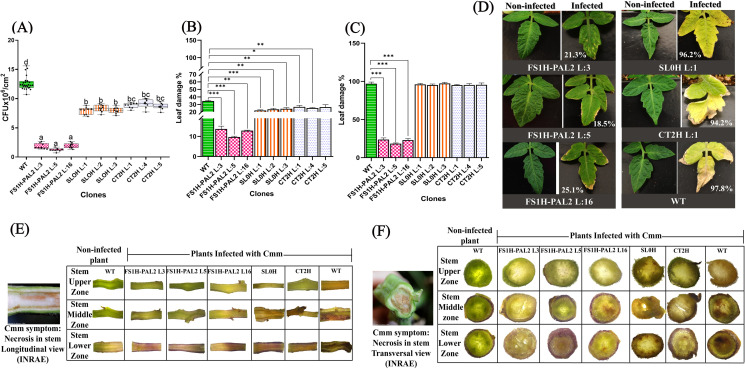
Colony forming units and lesion development in tomato plants. **(A)** Colony Forming Unit (CFU) count in leaves taken 9 days after Cmm infection. **(B)** Disease severity as percentage of leaf damage in tomato leaves 9 days after infection. **(C)** Disease severity as percentage of leaf damage in tomato leaves 120 days after infection. For **(A)**, a Tukey’s test was performed at α=0.01, with n = 3 biological replicates, each with three technical replicates (except for the WT line, for which n = 9 biological replicates with three technical replicates is shown); data represent mean ±  SD. For (B) and **(C)**, statistical analysis was performed from three biological replicates, using Welch’s one-way ANOVA followed by Dunnett’s multiple comparisons test. Statistically significant differences are indicated as follows: **p** <  0.005, * ; **p** <  0.01, **, **p** <  0.001, ***; **p** <  0.0001, ****; the absence of significance bars indicates that no significant (ns) differences were detected. **(D)** Phenotypes of non-infected and infected tomato leaves 120 days after infection. **(E)** Cmm symptomatology in upper, middle and lower stem regions from longitudinal sections from distinct infected tomato plants (reference image taken from INRAE [44]). **(F)** Cmm symptomatology in upper, middle and lower stem from transversal sections from distinct infected tomato plants (reference image taken from INRAE [44]).

Next, disease severity was assessed by measuring the total number of colony-forming units (CFUs) in leaves from plants exposed to the pathogen. As shown in Fig 7, nine days after infection (t_3_ =  9 dai), CFU counts were significantly lower in FS1H-PAL2 edited plants, ranging from 1.2 to 1.9 ×  10^6^ CFU/1 cm^2^ infected tissue, compared to control plants, where CFU counts were substantially higher (e.g., 7 to 9 ×  10^6^ CFU/1 cm^2^ in SL0H and CT2H, and approximately 1.3 ×  10^7^ CFU/1 cm^2^ in WT plants). Thus, bacterial populations in epigenetically edited plants exposed to the pathogen were more than an order of magnitude lower than in non-edited WT control plants. These findings showed that the epigenetically edited plants exhibited enhanced resistance to Cmm infection. Moreover, as previously observed, the percentage of lesion size in FSH1-PAL2 plants was markedly smaller than in all control plants (WT, CT2H and SL0H), whose lesion sizes ranged from 94.2% to 97.8% at 120 dai (Fig 7C-D).

**Fig 8:**
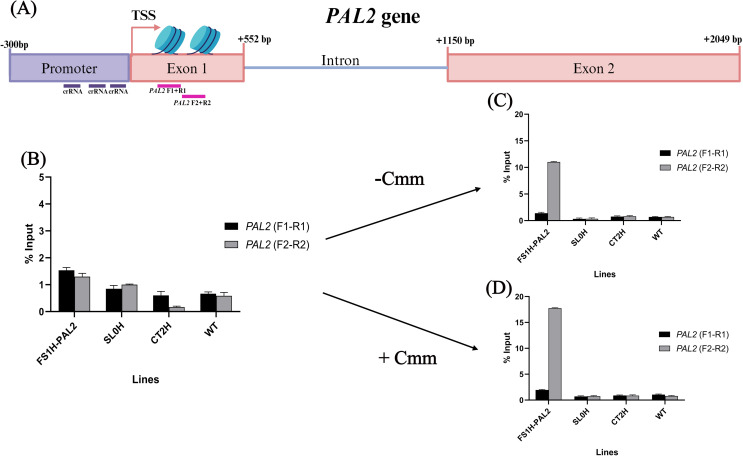
Histone methylation profile in tomato plants. Chromatin Immunoprecipitation (ChIP) assays to analyze the histone H3 lysine-4 trimethylation mark (H3K4me3) at the *SlPAL2* first exonic region. **(A)** Schematic representation of the *SlPAL2* gene targeted in the ChIP assay. The purple rectangle denotes the promoter region, red rectangles indicate exonic regions, purple lines show the crRNA binding sites, pink lines represent the regions amplified by PCR using two distinct sets of primers, and the bent arrow marks the transcription start site (TSS). **(B)** ChIP assays conducted on 21-day-old plants, 24 hours before infection with *Clavibacter michiganensis* subsp. *michiganensis* (Cmm). **(C)** ChIP assays from control plants that were not exposed to the pathogen (24-days-old plants). **(D)** ChIP assays performed 72 hours after Cmm infection. Data are mean ±  SEM. Each ChIP experiment was independently conducted in duplicate (two plants per line as technical replicates) from two biological replicates. ChIP-qPCR data were normalized to input samples (1% starting chromatin), according to the Percent Input method *(% Input* =  2^((Cq(IN)-Log^_2_^(DF))-Cq(IP))^ *  100; [40]).

We then investigated whether the increased *SlPAL2* expression and enhanced disease resistance were linked to changes in the chromatin structure of the *SlPAL2* gene. To explore this, we conducted chromatin immunoprecipitation (ChIP) assays to examine the status of the H3K4me3 histone mark at the first exonic region (putative + 1 and + 2 nucleosomes, located at + 45 to + 235 bp and + 238 to + 330 bp, respectively). We hypothesized that this region was being modified by the dCas12a-SET recombinant protein in FSH1-PAL2 plants, as the dCas12a-SET protein is guided to the *SlPAL2* promoter by the crRNAs (Fig 8A). ChIP assay results revealed that the enhanced resistance to Cmm was indeed associated with chromatin changes in the first exonic region of the *SlPAL2* gene. We determined that, prior to infection (t_0_ =  24 h before pathogen exposure), FS1H-PAL2 plants showed a slight 1-fold enrichment in the H3K4me3 histone activation mark at the 5′-end chromatin region (nucleosomes + 1 and + 2) compared to control plants (Fig 8B). This enrichment is likely due to the activity of the dCas12a-SET recombinant protein.

As the plants aged and transitioned from the vegetative to flowering stage (S5 Fig), non-infected FS1H-PAL2 plants exhibited an 11-fold increase in H3K4me3 enrichment, particularly in the + 238 to + 330 bp region (nucleosome + 2; Fig 8C). This enrichment, most likely deposited by the dCas12a-SET protein, correlated with increased *SlPAL2* expression. After Cmm infection, the H3K4me3 enrichment in FS1H-PAL2 plants further increased to 18-fold (Fig 8D), aligning with the elevated *SlPAL2* transcript levels (Fig 6B). These findings suggest a chromatin rearrangement in FS1H-PAL2 edited plants, positioning a phased nucleosome at + 238 to + 330 bp relative to the TSS (primer pair PAL2 F2 +  R2). This positioning likely facilitates transcription factor binding, enhancing gene expression [46], and may also help prevent transcription initiation from cryptic promoters [47,48]. The increased H3K4me3 enrichment in infected FS1H-PAL2 plants not only correlates with elevated *SlPAL2* expression but also with enhanced disease resistance.

**Fig 9:**
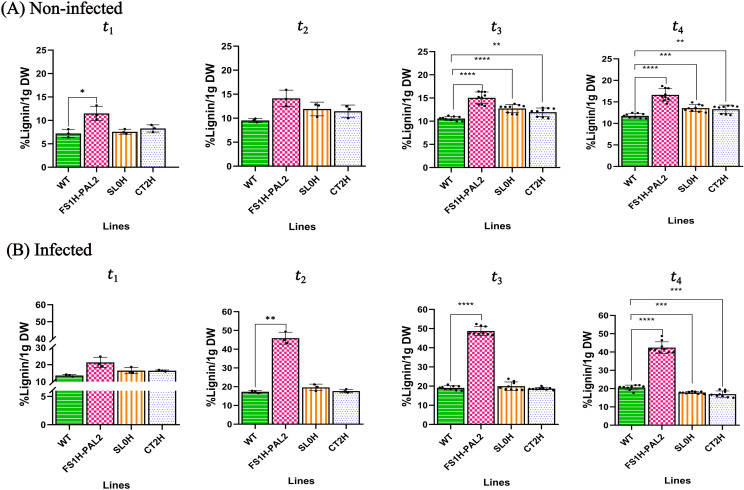
Lignin contents in edited tomato plants. Quantification of lignin contents in leaf tissue to assess the effect of *PAL2* gene activation on lignin deposition. **(A)** Non-infected plants (upper row) and (B) infected plants (lower row) at different time points: t_1_ (1-day post-infection, dpi), t_2_ (3 dpi), t_3_ (12 dpi) and t_4_ (120 dpi). Statistical analysis was performed using Welch’s one-way ANOVA followed by Dunnett’s multiple comparisons test, based on three biological replicates (n = 3), each with three technical replicates (for t_3_ and t_4_). Statistically significant differences are indicated as follows: **p** <  0.005, * ; **p** <  0.01, **, **p** <  0.001, ***; **p** <  0.0001, ****. Data represents mean ±  SEM. The absence of significance bars indicates that no significant (ns) differences were detected.

Similar to the first trial, lignin deposition was assessed as a defense response against Cmm. Lignin contents were measured in both non-infected and infected tomato plants at 1, 3, 12, and 120 days after infection, with non-infected plants sampled at the same time points (Fig 9). Results showed a statistically significant increase in lignin contents in non-infected FS1H-PAL2 edited plants at 22 days after germination (equivalent to 1 day after infection), with a 12% increase compared to control plants (SL0H: 7%, CT2H: 8%, and WT: 7%; Fig 9A). This initial rise in lignin contents can be attributed to the slightly elevated *SlPAL2* expression observed in non-infected FS1H-PAL2 plants. As the plants transitioned from the vegetative to the flowering stage, lignin deposition continued to increase moderately in non-infected FS1H-PAL2 plants, rising from 14% at 24 days (3 days after infection) to 16% at 141 days after germination (120 days after infection). While lignin levels also increased in control plants (SL0H, CT2H, and WT), the levels remained consistently lower than those in the edited plants. In contrast, Cmm-infected FS1H-PAL2 plants showed a marked increase in lignin contents following infection (Fig 9B), rising from 20% at 1 day after infection to 48% at 12 days, before slightly declining to 42% at 120 days (similar to the levels observed in the first trial; see Fig 5). In comparison, control plants exhibited only a modest increase in lignin content post-infection, reaching a maximum of 17.8%, 17%, and 20.6% (at 120 days) for SL0H, CT2H, and WT, respectively (Fig 9F). These results indicate that the increased lignin accumulation in Cmm-challenged FS1H-PAL2 plants is a robust defense response to biotic stress. Furthermore, this response correlates with elevated *SlPAL2* expression and enrichment of the H3K4me3 activation mark, supporting the role of *SlPAL2* in strengthening plant defenses.

**Fig 10:**
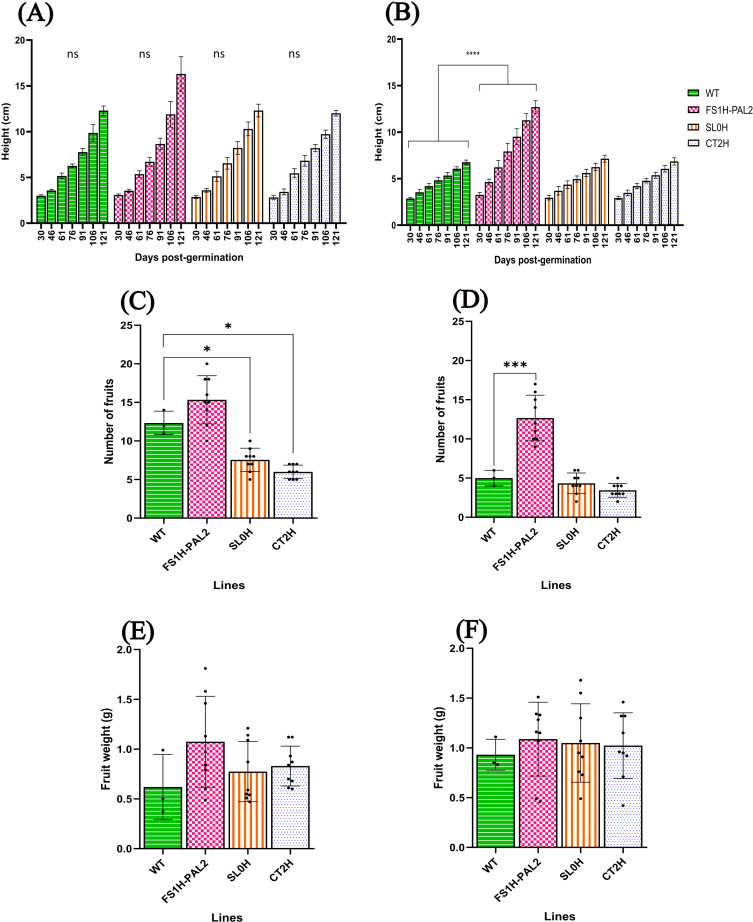
Agronomic characteristics in edited tomato plants. Plant height at different time points (days post-germination): (A) non-infected plants, (B) plants infected with Cmm. Number of fruits: (C) non-infected plants, (D) plants infected with Cmm. Fruit weight: (E) non-infected plants, (F) plants infected with Cmm. For A-B, statistical analysis was performed using two-way ANOVA followed by Tukey’s multiple comparisons test (n = 3 biological replicates). Statistically significant differences are indicated as follows: **p** <  0.005, * ; **p** <  0.01, **, **p** <  0.001, ***; **p** <  0.0001, ****); data represents mean ±  SD. For C-F, statistical analysis was performed using Welch’s one-way ANOVA followed by Dunnett’s multiple comparisons test (n = 3 biological replicates, each with three technical replicates, except for the WT line for which n = 3 biological replicates). Statistically significant differences are indicated as follows: **p** <  0.005, * ; **p** <  0.01, **, **p** <  0.001, ***; **p** <  0.0001, ****. The absence of significance bars indicates that no significant (ns) differences were detected.

As performed in the first trial, we also evaluated plant height, fruit numbers, and fruit weight in both infected and non-infected plants, to assess the effect of CRISPRa editing on the indicated agronomic traits. As shown in Fig 10A, there were no statistically significant differences in plant height among non-infected lines. Nonetheless, after infection, control plants showed reduced height (Fig 10B), while FS1H-PAL2 plants continued to grow normally. Remarkably, non-infected FS1H-PAL2 plants produced more fruits per plant compared to SL0H and CT2H control plants (Fig 10C). Although fruit productivity declined across all lines following Cmm infection, all infected control plants still had significantly fewer fruits than FS1H-PAL2 edited plants (Fig 10D). In comparison, there were no statistically significant changes in fruit weight between infected and non-infected plants, nor among the different lines (Fig 10E–F).

## Discussion

Phenylalanine ammonia-lyase (PAL) is a key enzyme in the phenylpropanoid pathway, which serves as the initial step in the production of numerous secondary metabolites. These include lignin, lignans, and flavonoids—compounds that are essential for plant structural integrity and defense [45]. Hence, PAL catalyzes the deamination of phenylalanine to cinnamic acid, leading to the synthesis of phenylpropanoids, which are critical for lignin biosynthesis. Lignin is not only essential for strengthening the plant cell wall and maintaining vascular integrity but also plays a crucial role in plant defense by inhibiting pathogen invasion [15,49]. By forming a physical barrier, lignin obstructs pathogen entry and limits the spread of toxins. Also, in response to various pathogen attacks, lignin deposition and the activation of genes involved in its biosynthesis are widely observed [50]. Several studies have examined the role of PAL in plants. For instance, the overexpression of a heterologous *PAL* gene from beans in tobacco plants triggered gene silencing, leading to reduced PAL activity and decreased phenylpropanoid production. This caused visible changes, such as altered leaf morphology, reduced lignification, stunted growth, and impaired flower development [51]. In a related study, transgenic tobacco plants with suppressed PAL levels exhibited increased susceptibility to fungal infections, developing more severe lesions after infection with *Cercospora nicotianae* [52]. Similar results have been observed in *Arabidopsis*, where plants with mutations in two *PAL* genes (*pal1 pal2* double mutants) displayed defects in tannin and anthocyanin biosynthesis and increased UV-B sensitivity, whereas quadruple mutants (*pal1 pal2 pal3 pal4*) exhibited severe developmental abnormalities, such as sterility and stunted growth, due to reduced lignin levels and heightened stress susceptibility [53]. Furthermore, in the resistant melon cultivar Ultrasweet Miami, inoculation with *Colletotrichum lagenarium* rapidly triggers localized defense responses, with the *PAL* gene playing a key role. Within 48 hours post-inoculation, PAL activity significantly increases the deposition of phenolic compounds and lignin around infection sites, reinforcing the cell wall and restricting pathogen spread [54]. Similarly, resistant pigeon pea (*Cajanus cajan* (L.) Millsp.) cultivars infected with *Fusarium udum*, the pathogen responsible for wilt disease in pigeon pea, showed significantly higher expression of the *PAL2* gene at all stages of infection compared to susceptible cultivars [55]. This suggests that resistant cultivars may reinforce their cell walls at the infection site through lignification and/or suberization, effectively eliminating the pathogen with phenols, phytoalexins, and other metabolites produced via the phenylpropanoid pathway. In contrast, susceptible cultivars (with lower *PAL2* gene expression) are unable to mount a sufficient defense, allowing *F. udum* to infect and colonize the plant [55]. These findings underscore the importance of the phenylpropanoid pathway in both plant development and defense.

The accumulation of lignin in response to pathogenic infection is well-documented. Lignin biosynthesis is upregulated in infected tissues, creating a physical barrier that prevents pathogen entry and spread [56–58]. This is accompanied by an increase in the activity of enzymes involved in the phenylpropanoid pathway, including PAL [49]. Lignin not only fortifies plant tissues but also plays a significant role in facilitating water transport and maintaining structural integrity, particularly in response to both biotic and abiotic stresses [15,59]. Conversely, studies focused on enhancing PAL activity have demonstrated that increasing phenylpropanoid production can bolster plant defenses. For example, transgenic tobacco plants overexpressing *PAL* exhibited higher levels of hydroxycinnamic acid esters and reduced susceptibility to *Cercospora nicotianae* infection [60]. In bamboo (*Phyllostachys edulis*), transient overexpression of *PAL* led to elevated lignin and flavonoid levels, further supporting the role of PAL in defense [61]. Similarly, overexpression of *RgPALs* in *Rehmannia glutinosa* enhanced phenolic production and played a role in resistance to replanting diseases, underscoring the connection between PAL and the phenylpropanoid pathway [62].

Despite these advances, there are currently no commercially available tomato (*S. lycopersicum*) lines that are naturally resistant to Cmm, or with high *PAL2* gene expression. Furthermore, studies on the epigenetic regulation of *PAL* in enhancing disease resistance are still absent. This research aimed to explore the use of CRISPRa (CRISPR activation) to epigenetically edit tomato plants by targeting the *SlPAL2* gene, with the goal of increasing lignin production and improving pathogen resistance. The advantage of using CRISPRa is that the target DNA sequence will not be changed. Therefore, tomato explants were transformed using biolistics with CRISPRa constructs designed to upregulate *SlPAL2* gene expression. The obtained edited tomato plants were evaluated for lignin content, disease resistance, and plant growth under greenhouse conditions.

Initial experiments showed that CRISPRa-edited plants exhibited increased *SlPAL2* transcription and demonstrated that the CRISPR-dCas12a system can be effectively used for activation of plant defense genes. In non-infected edited plants, *SlPAL2* expression rose 2.5-fold, peaking at t_2,_ before gradually decreasing. Upon infection with Cmm, the bacterial strain responsible for bacterial canker, *SlPAL2* expression increased 3.9-fold at t_2_ (3 dai). In contrast, *SlPAL2* expression in all control plants practically remained unchanged after infection. This heightened expression was associated with a marked reduction in pathogen load (as measured by CFUs) and lesion size in edited plants compared to controls. Our results are consistent with the hypothesis that directed CRISPRa to the promoter region of the *SlPAL2* gene enhanced its transcriptional activation and protected tomato plants against severe Cmm infection. In addition, we determined that height and fruit yield was not compromised in the epigenetically edited plants, and that solely control plants showed a stunted growth after Cmm infection. Although the number of fruits was slightly reduced after infection in control and edited plants. Furthermore, lignin contents in infected FS1H-PAL2 plants increased around 2.5-fold, 120 days after germination. These results confirm that activating *SlPAL2* through CRISPRa not only enhances lignin biosynthesis but also strengthens the plant’s defense against Cmm infection. Consequently, enhancing plant disease resistance via spatial and temporal control of lignin biosynthesis presents a promising approach to protect crops against pathogens while maintaining or markedly improving their productivity [43,49,63].

An experimental biological replication was performed to verify and expand our findings. As shown, in non-infected edited plants *SlPAL2* expression rose 2-fold, as an average, particularly at t_2_, and then transcript levels decreased with time. Upon infection with the pathogen, *SlPAL2* expression increased 4-fold at t_2_ (3 dai), before gradually decreasing. In contrast, *SlPAL2* expression in all control plants practically remained unchanged after infection. Disease evaluation was ascertained by examining the total number of CFUs and lesion size in infected plants. As indicated, the number of CFUs in unedited control plants was over one order of magnitud than the number of CFUs found in FSH1-PAL2 locally infected leaves, whereas the percentage of lesion size in edited plants was much lower than in all control plants. Thus, epigenetically edited plants showed increased *SlPAL2* expression, presented a substantial reduction of disease symptoms, and exhibited enhanced resistance to Cmm.

Further chromatin immunoprecipitation (ChIP) assays provided insights into the epigenetic changes associated with *SlPAL2* activation. Edited plants showed increased enrichment of the H3K4me3 activation mark at the *SlPAL2* locus, correlating with higher gene expression and pathogen resistance. These findings suggest that CRISPRa-mediated epigenetic modifications can effectively enhance plant defense by establishing a transcriptionally active chromatin state at key defense gene loci. Additionally, lignin contents show a relationship between *SlPAL2* expression, H3K4me3 enrichment, and disease resistance. Interestingly, while *SlPAL2* expression declined over time after pathogen infection, the increased lignin content in edited plants remained stable, suggesting that the enhanced lignin deposition was sufficient to maintain plant structural integrity and defense capacity. This aligns with previous findings that PAL and other phenylpropanoid pathway enzymes are subject to complex regulation, including transcriptional, post-translational, and both feedforward and feedback regulatory mechanisms [43,64]. Moreover, the elevated lignin content in edited plants may serve as a scaffold for sustained resistance, even when *SlPAL2* expression diminishes, as it occurs in the pathosystem *S. tuberosum - Rhizoctonia solani* [43]. Thus, increased PAL content in edited plants could affect the biosynthesis of downstream metabolites which leads to increased resistance to pathogen infection [65], and to the biosynthesis of metabolic derivatives that inhibit or regulate the phenylalanine ammonia-lyase production or activity [64–66], even in CRISPRa plant. Transcriptomic, metabolomic and proteomic assays will certainly provide a broader understanding of the edited plant’s defense responses. In addition, it would be valuable to assess whether *SlPAL2* activation, enhanced resistance and chromatin modifications persist across multiple generations, offering valuable insights into the stability and heritability of these traits.

Overall, *SlPAL2* gene expression in edited plants did not lead to toxicity effects [67]. Furthermore, the strategy used here avoids having a second copy of the target gene present, which avoids the possibility of triggering gene silencing mechanisms as has been shown before [51,52]. Ectopic *SlPAL2* gene expression was attained by making use of the cell’s native apparatus to upregulate target endogenous gene expression levels, edited plants presented enhanced disease resistance and higher lignin contents, and did not show a negative impact on crop productivity. Thus, CRISPRa is a promising tool to improve agriculturally important traits in crops. Whereas traditional breeding depends on existing genetic variation within a population and often requires several generations to select and stabilize desirable traits (which can take years), CRISPRa can achieve desired gene activation in a single generation and can create novel phenotypes by activating silent or less-expressed genes, significantly accelerating the development process. In addition, CRISPRa can activate multiple genes or entire pathways simultaneously, enabling complex trait improvement that would be hard to achieve with traditional breeding.

Beyond disease resistance, the potential applications of plants with increased lignin production extend to other areas of agriculture. For instance, as a complex phenolic polymer, lignin reinforces the plant cell wall, providing structural rigidity, enhancing hydrophobicity, and facilitating the efficient transport of minerals through vascular tissues. In addition, lignin metabolism is actively involved in plant lodging resistance and responses to environmental stresses, such as drought or salinity. Consequently, understanding lignin biosynthesis and its function not only advances agricultural productivity but also has broader implications for sustainable industry and human applications [70]. For example, lignin-derived nanomaterials, such as lignin nanoparticles (LN), have shown promise as biostimulants (nanobiostimulants), improving plant growth and stress tolerance [68]. Accordingly, plant biostimulants when applied to plants or the rhizosphere stimulate natural processes to benefit nutrient uptake and efficiency, tolerance to abiotic stress, and/or crop quality [69]. Furthermore, an important supply of biostimulants are organic waste streams, positioning these products in the limelight for innovation in agriculture [71]. By enhancing lignin biosynthesis, CRISPRa-edited plants could also contribute to the sustainable production of nanobiostimulants, which promote nutrient uptake and improve crop quality [71]. Consequently, CRISPRa and green nanotechnology offer benefits for use in sustainable agricultural production practices.

Epigenome editing, particularly using CRISPRa [29,33,72], provides a powerful tool to enhance disease resistance in crops without negatively affecting growth or productivity. By targeting key defense genes such as *SlPAL2*, plants can be engineered to exhibit increased lignin content and enhanced pathogen resistance. For epigenetically edited plants to be classified as non-genetically modified organisms (non-GMOs), however, it is essential that no plasmid DNA sequences remain in the genome of the final product. Thus, to further advance on CRISPRa as an alternative to conventional plant breeding practices, once the T-DNA has been segregated out in the progeny, it becomes critical to assess whether the introduced epigenetic marks are mitotically and/or meiotically stable, if the native epigenetic marks are restored once the epigenome editor is removed, and/or if the induced epialleles could serve as valuable sources of novel trait variation, enabling the breeding of plants with unique phenotypes and new traits [72]. This research demonstrates the potential of CRISPRa (using dCas12a), to activate endogenous defense mechanisms and offers new opportunities for sustainable agricultural practices.

## Supporting information

S1 Fig Stepwise procedure for vector generation. (A) The PAL2 crArray was cloned into the BsaI restriction sites of (B) the p143-L2 vector. This resulted in (C) the p143L2 + PAL2 crArray vector, which was used as one of the entry vectors for Gateway LR Cloning, along with (D) the p233-SETX vector (containing the catalytic SET domain of ATX1 fused to the C-terminus of dCas12a). (E) The CT2H vector (p203-GFP-Hyg) served as the destination vector, leading to the final product: (F) the expression vector FS1H-PAL2 (dCas12 + SET+crRNA-PAL2). Following a similar approach, the control vector (G) SL0H (dCas12 + SET+ΔcrRNA) was obtained through the recombination of (B), (D), and (E). (H) Confirmation of the FS1H-PAL2 construct (dCas12 + SET+crRNA PAL2) by restriction analysis using the enzymes (1) EcoRI and (2) HindIII. MW: Molecular weight marker (Thermo Scientific™ O’GeneRuler 1 kb Plus DNA Ladder, Ready-to-Use, catalog #SM1343).
(TIF)

S2 Fig Chromatin digestion and specificity of ChIP primers. (A) Chromatin digestion of edited and control, non-infected and infected plants, using 1U Micrococcal Nuclease (MNase, Thermo Scientific cat. #88216) at 37°C for 25 min. (B) PCR amplification using digested chromatin as template, to validate the specificity of primers used for ChIP-qPCR. Figure shows the efficient amplification of DNA fragments corresponding to the two putative nucleosomes positioned within exon 1, demonstrating that both sets of primers effectively amplify the target sequences. MW1: GeneRuler 100 bp DNA Ladder, Thermo Scientific® catalog #SM0241; MW2: 10 bp DNA Ladder, Invitrogen® catalog #10821-015; NTC: non-template control; C + : genomic DNA.
(TIF)

S3 Fig Genotyping of *SlPAL2* edited plants. PCR amplification of the CaMV35S promoter fragment (1.26 kb) or the dCas12 + SET+crRNA+Attb2 region (2.5 kb) in FS1H-PAL2 edited plants.
(A-B) PCR amplification of the CaMV35S promoter region and (C-D) PCR amplification of dCas12 region from single parental epigenetically edited tomato plants. MW: Molecular weight marker (Thermo Scientific™ O’GeneRuler 1 kb Plus DNA Ladder, Ready-to-Use, catalog: SM1343). FS1H-PAL2: plants transformed with dCas12 + SET+crRNA-PAL2; SL0H: plants transformed with dCas12 + SET+ΔcrRNA; CT2H: plants transformed with p203 + GFP+Hyg; C + : positive control (FS1H-PAL2 vector construct); NTC: no template control; WT: wild-type plants. Numbers correspond to individual independent clones from each line.
(TIF)

S4 Fig Genotyping of *SlPAL2* edited T1 plants. PCR amplification of the CaMV35S promoter fragment (1.6 kb) from single T1 epigenetically edited tomato plants. MW: Molecular weight marker (NEB Quick-Load® DNA Marker, Broad Range catalog #N0303). FS1H-PAL2: plants transformed with dCas12 + SET+crRNA-PAL2; SL0H: plants transformed with dCas12 + SET+ΔcrRNA; CT2H: plants transformed with p203 + GFP+Hyg; WT: wild-type plants; NTC: no template control; C + : positive control (FS1H-PAL2 vector construct). Numbers correspond to individual independent clones from each line.
(TIF)

S5 Fig Number of flowers per plant at different times post-germination. (TIF)

S1 Table crRNA guide design and computational prediction of possible crRNA off-targets projected with CHOPCHOP v3, CRISPR-P v2.0 and Cas-OFFinder tool [73].
(DOCX)

S2 Table DNA Sequences and description of vectors used in this study.
(TIF)

S3 Table Experimental design of Cmm infection assays. FS1H-PAL2 (dCas12 + SET + 3crRNA PAL2), SL0H (dCas12 + SET - ΔcrRNA), CT2H (p203-GFP-Hyg empty vector), WT (wild type Micro-Tom).
(TIF)
