# Supplementary figures and images for: CRISPR/dCas12a-mediated activation of SlPAL2 enhances tomato resistance against bacterial canker disease
Source: PLoS One. 2025 Mar 26;20(3):e0320436. doi: 10.1371/journal.pone.0320436 (PMC11940823; doi:10.1371/journal.pone.0320436)

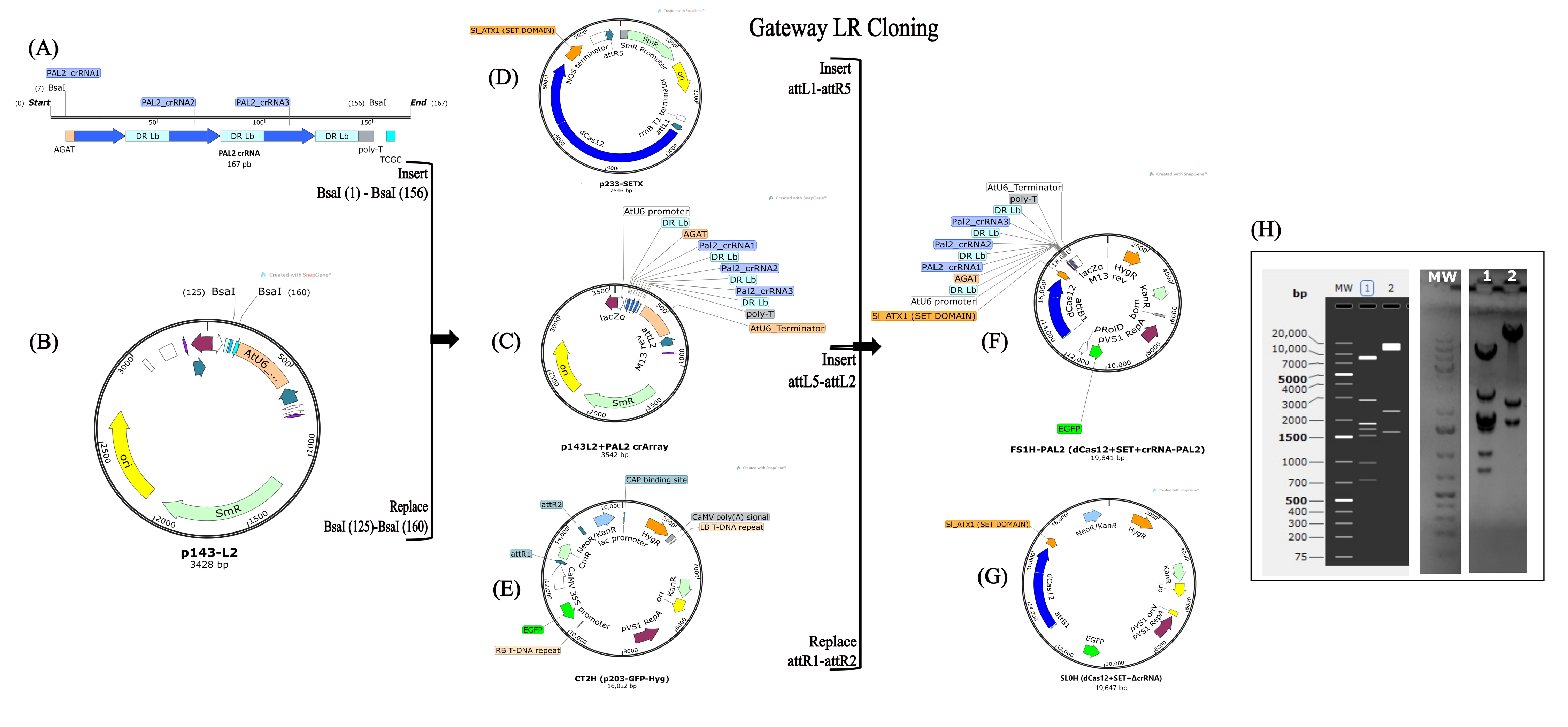

Supplement: S1 Fig — This resulted in (C) the p143L2 + PAL2 crArray vector, which was used as one of the entry vectors for Gateway LR Cloning, along with (D) the p233-SETX vector (containing the catalytic SET domain of ATX1 fused to the C-terminus of dCas12a). (E) The CT2H vector (p203-GFP-Hyg) served as the destination vector, leading to the final product: (F) the expression vector FS1H-PAL2 (dCas12 + SET+crRNA-PAL2). Following a similar approach, the control vector (G) SL0H (dCas12 + SET+ΔcrRNA) was obtained through the recombination of (B), (D), and (E). (H) Confirmation of the FS1H-PAL2 construct (dCas12 + SET+crRNA PAL2) by restriction analysis using the enzymes (1) EcoRI and (2) HindIII. MW: Molecular weight marker (Thermo Scientific™ O’GeneRuler 1 kb Plus DNA Ladder, Ready-to-Use, catalog #SM1343). (TIF) [file pone.0320436.s001.tif]

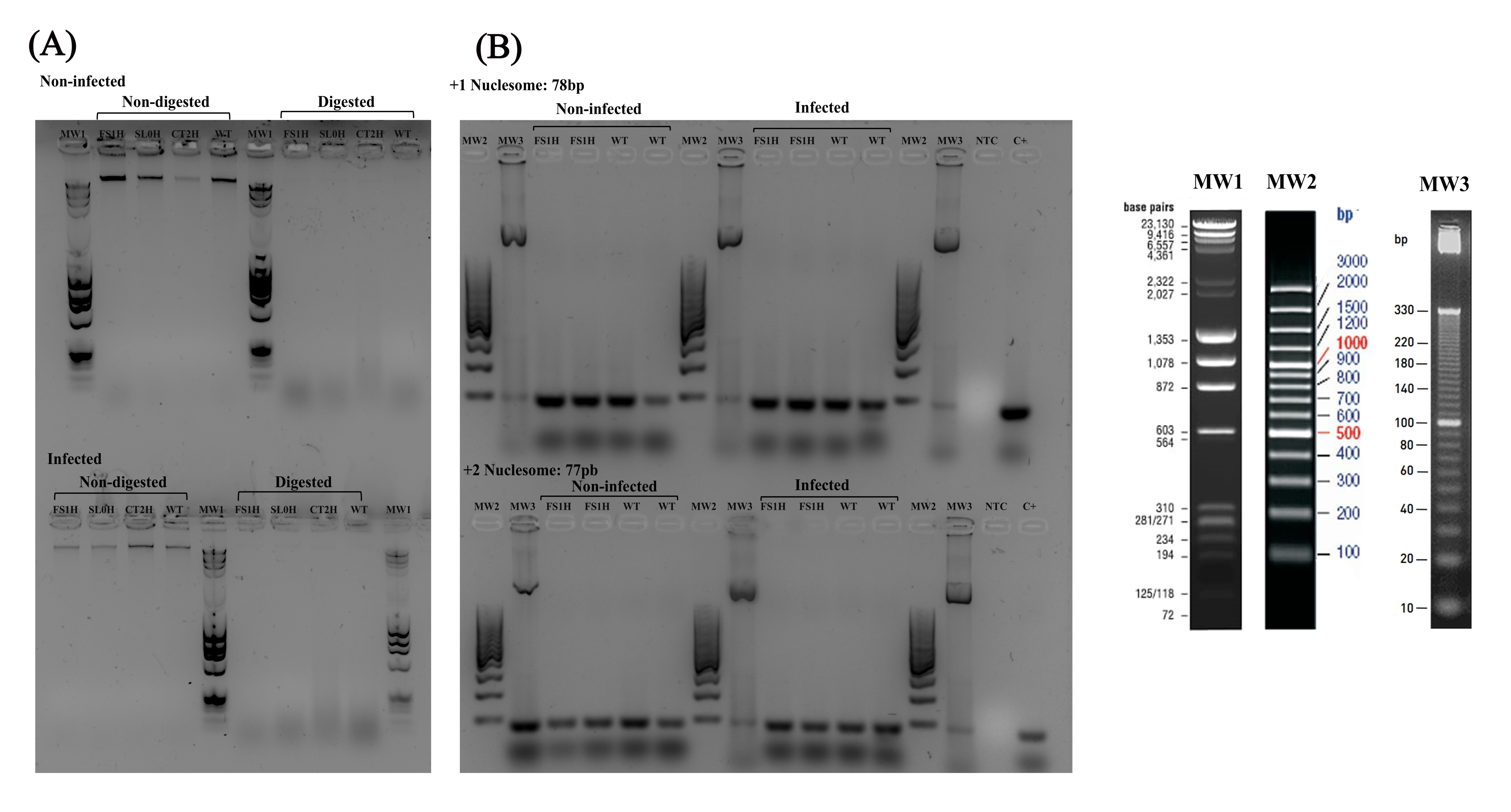

Supplement: S2 Fig — #88216) at 37°C for 25 min. (B) PCR amplification using digested chromatin as template, to validate the specificity of primers used for ChIP-qPCR. Figure shows the efficient amplification of DNA fragments corresponding to the two putative nucleosomes positioned within exon 1, demonstrating that both sets of primers effectively amplify the target sequences. MW1: GeneRuler 100 bp DNA Ladder, Thermo Scientific® catalog #SM0241; MW2: 10 bp DNA Ladder, Invitrogen® catalog #10821-015; NTC: non-template control; C + : genomic DNA. (TIF) [file pone.0320436.s002.tif]

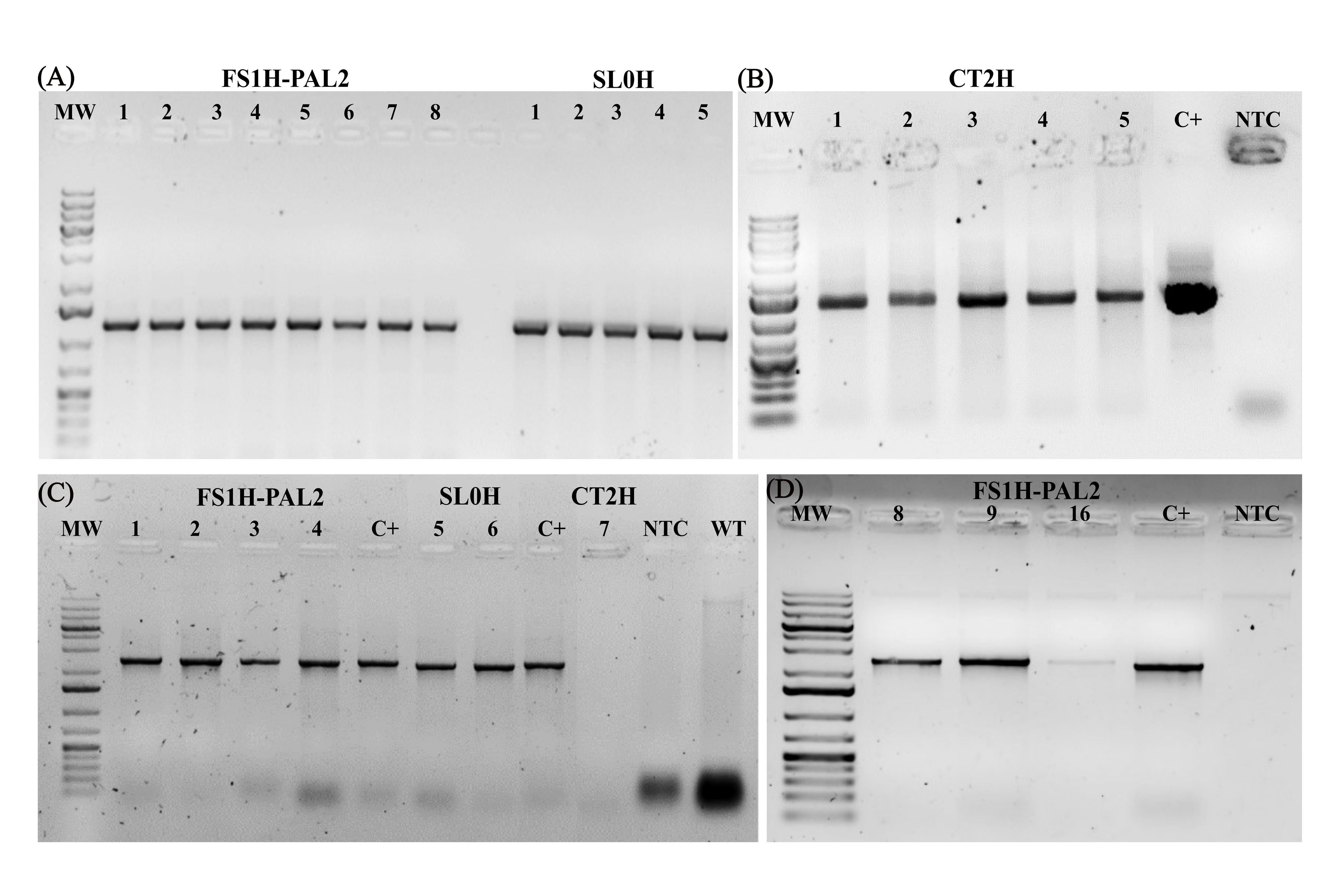

Supplement: S3 Fig — (A-B) PCR amplification of the CaMV35S promoter region and (C-D) PCR amplification of dCas12 region from single parental epigenetically edited tomato plants. MW: Molecular weight marker (Thermo Scientific™ O’GeneRuler 1 kb Plus DNA Ladder, Ready-to-Use, catalog: SM1343). FS1H-PAL2: plants transformed with dCas12 + SET+crRNA-PAL2; SL0H: plants transformed with dCas12 + SET+ΔcrRNA; CT2H: plants transformed with p203 + GFP+Hyg; C + : positive control (FS1H-PAL2 vector construct); NTC: no template control; WT: wild-type plants. Numbers correspond to individual independent clones from each line. (TIF) [file pone.0320436.s003.tif]

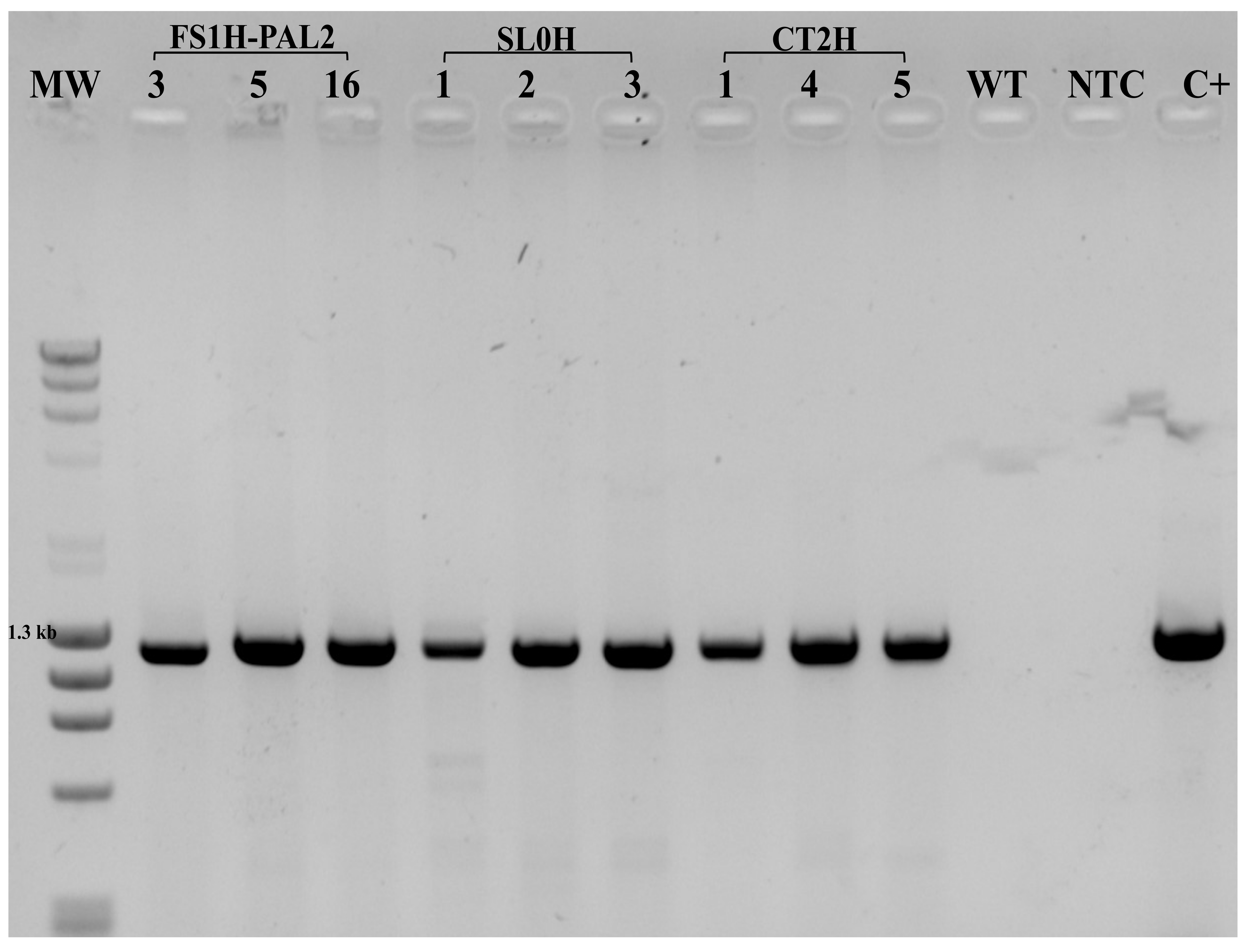

Supplement: S4 Fig — PCR amplification of the CaMV35S promoter fragment (1.6 kb) from single T1 epigenetically edited tomato plants. MW: Molecular weight marker (NEB Quick-Load® DNA Marker, Broad Range catalog #N0303). FS1H-PAL2: plants transformed with dCas12 + SET+crRNA-PAL2; SL0H: plants transformed with dCas12 + SET+ΔcrRNA; CT2H: plants transformed with p203 + GFP+Hyg; WT: wild-type plants; NTC: no template control; C + : positive control (FS1H-PAL2 vector construct). Numbers correspond to individual independent clones from each line. (TIF) [file pone.0320436.s004.tif]

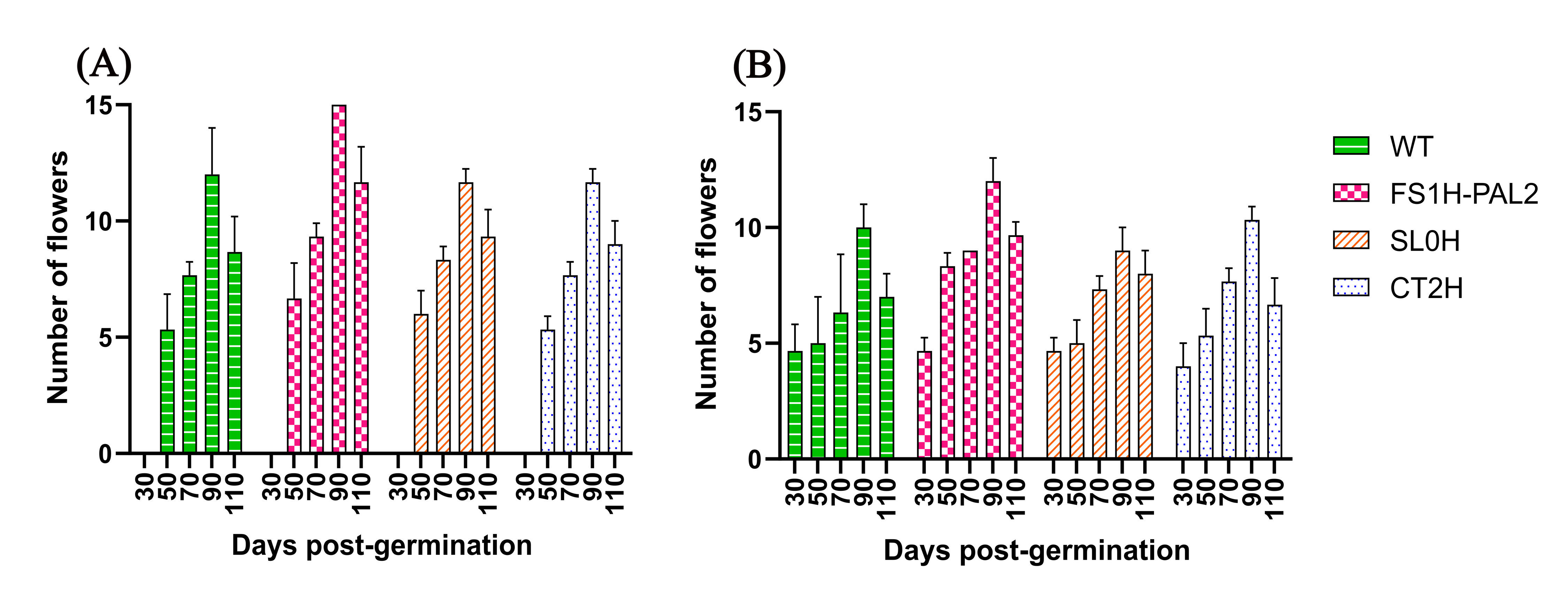

Supplement: S5 Fig — (TIF) [file pone.0320436.s005.tif]

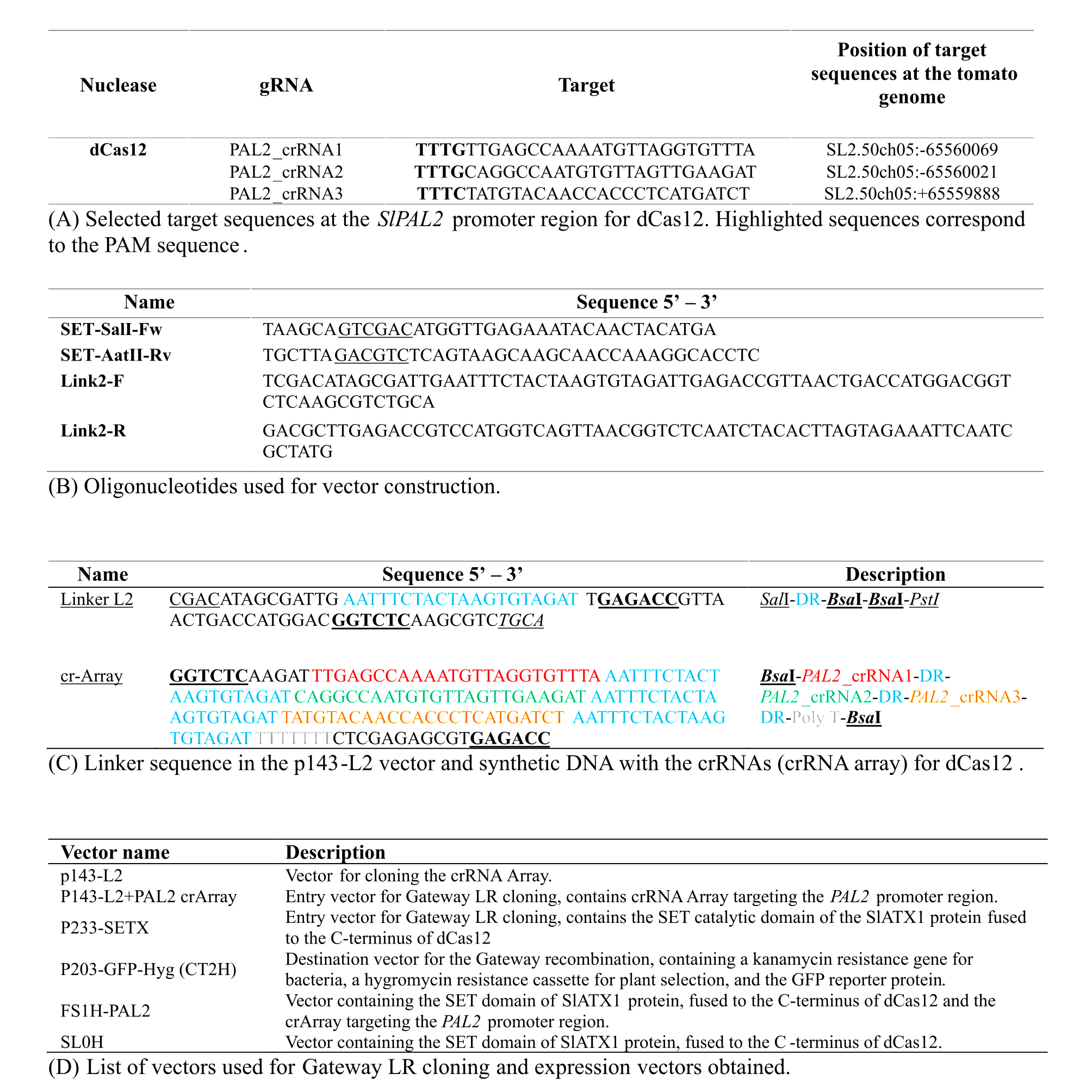

Supplement: S2 Table — (TIF) [file pone.0320436.s007.tif]

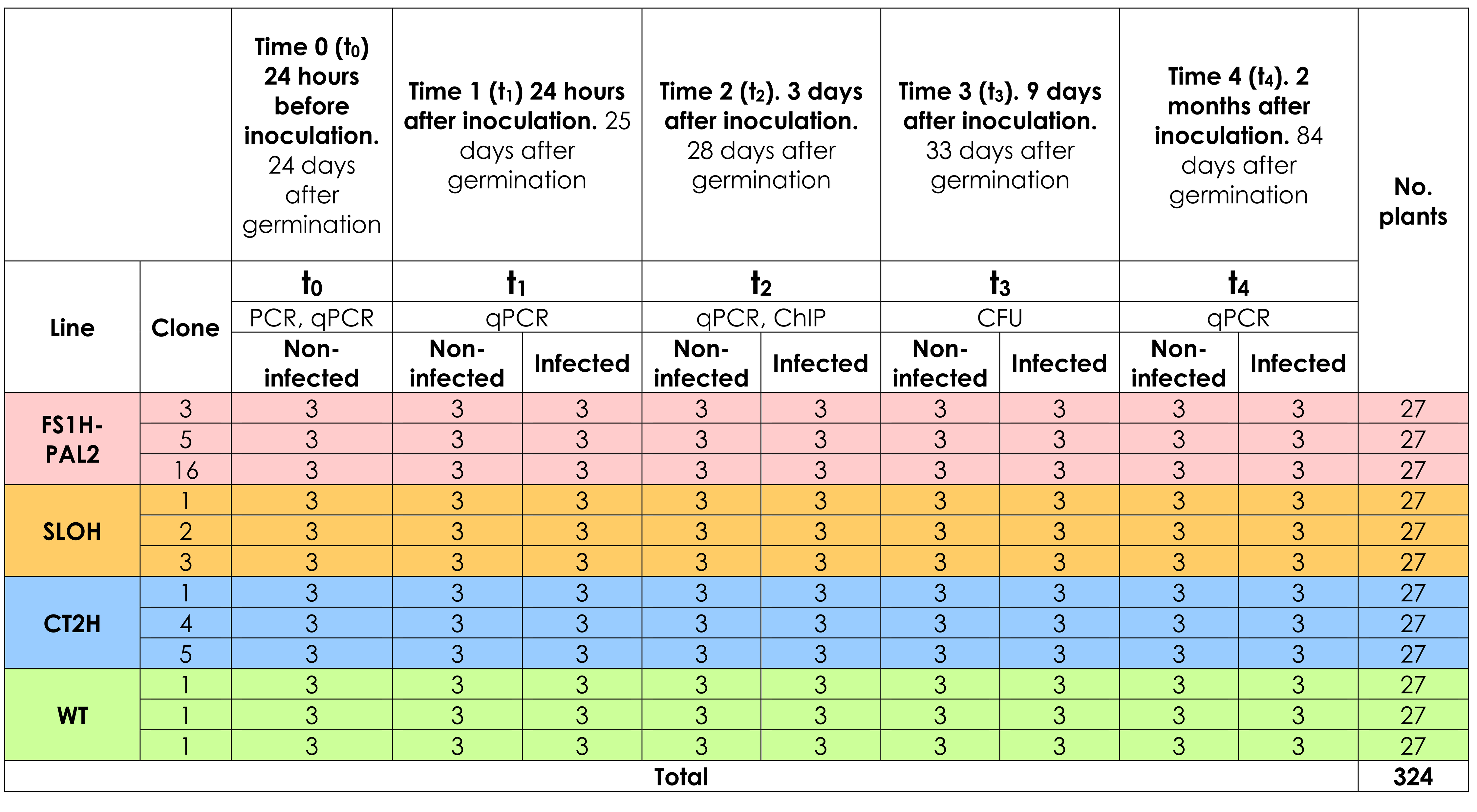

Supplement: S3 Table — FS1H-PAL2 (dCas12 + SET + 3crRNA PAL2), SL0H (dCas12 + SET - ΔcrRNA), CT2H (p203-GFP-Hyg empty vector), WT (wild type Micro-Tom). (TIF) [file pone.0320436.s008.tif]
