# Supplementary material for: CRISPR/dCas12a-mediated activation of SlPAL2 enhances tomato resistance against bacterial canker disease
Source: PLoS One. 2025 Mar 26;20(3):e0320436. doi: 10.1371/journal.pone.0320436 (PMC11940823; doi:10.1371/journal.pone.0320436)
Supplement: S1 Table — (DOCX) [file pone.0320436.s006.docx]

S1 Table. crRNA guide design and computational prediction of possible crRNA off-targets projected with CHOPCHOP v3, CRISPR-P v2.0 and Cas-OFFinder tool.

| crRNA sequence (5’-3’) | Number of off-targets predicted | | |
| --- | --- | --- | --- |
|  | CHOPCHOP v3 | CRISPR-P v2.0 | Cas-OFFinder tool |
| TTTCTATGTACAACCACCCTCATGATCT | 0 | NP | 0 |
| TTTGTTGAGCCAAAATGTTAGGTGTTTA | NP | 0 | 0 |
| TTTGCAGGCCAATGTGTTAGTTGAAGAT | NP | 0 | 0 |

NP: not predicted. These guides were not predicted using the specified tool.

First, for crRNA guide design, a 1000 bp region upstream of the *SlPAL2* gene coding sequence in *Solanum lycopersicum* (Gene ID: 101249824, GenBank accession: NM_001320601, Solgenomics: Solyc05g056170.2) was selected for crRNA design (putative promoter region). Next, candidate crRNAs for CRISPR/dCas12a (LbCpf1, PAM: 5’-TTTV-3’) binding were identified using online tools such as CRISPR-P 2.0 [30] and CHOPCHOP [31]. Three CRISPR RNAs (crRNAs) were chosen based on their location within the first 300 bp upstream of the transcription start site (TSS), ensuring that no cis-regulatory elements—such as the TATA and CAAT boxes, or the TSS itself—were disrupted and with minimal off-target potential, by using the following tools:

- Guide Design Using CHOPCHOP v3 settings:
- Target: The promoter sequence of *SlPAL2* (up to 300 bp upstream of the transcription start site [TSS]) was pasted.
- In: The tomato genome *Solanum lycopersicum* SL3.0 was selected as the target reference.
- Using: CRISPR/Cpf1 or CasX was chosen.
- For: Activation

After configuring these parameters, the tool was executed. CHOPCHOP v3 uses a color-coded scoring system for guide visualization: green: high-quality guides; yellow: moderate-quality guides; red: low-quality guides; finally, this tool also indicates the possibility of off-target.

- Guide Design Using CRISPR-P v2.0 settings:
- PAM Sequence: TTTV (AsCpf1 from Acidaminococcus or LbCpf1 from Lachnospiraceae: 5'-TTTV-3') was selected
- Promoter: The U6 promoter was selected.
- RNA Scaffold: Default settings.
- Guide Length: 20 nucleotides.
- Target Genome: *Solanum lycopersicum* (SL 3.0).
- Locus Tag: empty.
- Position: empty.
- Sequence: The sequence of the promoter region of SlPAL2 was pasted.

After submitting the input, CRISPR-P v2.0 generated potential guides, accompanied by a color-coded scoring system: green: high-quality guides;gray: moderate-quality guides; red: low-quality guides, also this tool indicates the possibility of off-target.

- Off-Target Search with Cas-OFFinder tool:

To ensure that the designed crRNAs did not have unpredicted off-target effects, an additional analysis was conducted using the Cas-OFFinder tool (<http://www.rgenome.net/cas-offinder/>), a fast and versatile algorithm for identifying potential off-target sites for RNA-guided endonucleases [73]. The following configurations were applied during the analysis:

- PAM Type: CRISPR/Cas-derived RNA-guided endonucleases (RGENs) were specified as: AsCpf1 from Acidaminococcus or LbCpf1 from Lachnospiraceae: 5'-TTTV-3' (V=G, C or A)
- Target Genome, Organism Type: Plants.
- Genome: *Solanum lycopersicum* (SL2.5) – Tomato.
- Mismatch Number: 0 to 3 (0, 1, 2, or 3 possible mismatches were selected, and the analysis repeated with each option)
- DNA Bulge Size: 0
- RNA Bulge Size: 0
- Query Sequences: Each crRNA sequence was used as input into the Cas-OFFinder tool without including the PAM sequence.

After configuring the parameters, the sequences were submitted for analysis. The output of all potential off-target sites for the input sequences was as follows:

**Summary**

Target Sequence Bulge Type Bulge Size Mismatch Number of Found Targets

TTTVCAGGCCAATGTGTTAGTTGAAGAT X 0 3 (0 to 3) 1

TTTVTATGTACAACCACCCTCATGATCT X 0 3 (0 to 3) 1

TTTVTTGAGCCAAAATGTTAGGTGTTTA X 0 3 (0 to 3) 1

For this study, only crRNAs with zero predicted off-targets were selected. This was verified by re-analyzing the crRNAs using the same Cas-OFFinder parameters to confirm their specificity:

**Details**

(0, 1, 2, or 3 possible mismatches were selected, and the analysis repeated with each option)

**Filter Download filtered result**

Bulge Type Target Chromosome Position Direction Mismatches Bulge Size

X crRNA: TTTVTTGAGCCAAAATGTTAGGTGTTTA

DNA: TTTGTTGAGCCAAAATGTTAGGTGTTTA chr5 65560044 - 3 0

X crRNA: TTTVCAGGCCAATGTGTTAGTTGAAGAT

DNA: TTTGCAGGCCAATGTGTTAGTTGAAGAT chr5 65559996 - 3 0

X crRNA: TTTVTATGTACAACCACCCTCATGATCT

DNA: TTTCTATGTACAACCACCCTCATGATCT chr5 65559911 + 3 0

**All bioinformatic tools indicate that the selected crRNA sequences had no predicted potential off-targets.**
